# Boronate‐Based Fluorescence Probes for the Detection of Hydrogen Peroxide

**DOI:** 10.1002/open.201700189

**Published:** 2018-01-26

**Authors:** Emma V. Lampard, Adam C. Sedgwick, Xiaolong Sun, Katherine L. Filer, Samantha C. Hewins, Gyoungmi Kim, Juyoung Yoon, Steven D. Bull, Tony D. James

**Affiliations:** ^1^ Department of Chemistry University of Bath BA2 7AY Bath UK; ^2^ Department of Chemistry The University of Texas at Austin Austin 78712 TX USA; ^3^ Department of Chemistry and Nanoscience Ewha Womans University Seoul 120–750 Korea

**Keywords:** boronic acids, diagnostics, fluorescent probes, H_2_O_2_, intramolecular charge transfer (ICT)

## Abstract

In this work, we synthesized a series of boronate ester fluorescence probes (*E*)‐4,4,5,5‐tetramethyl‐2‐(4‐styrylphenyl)‐1,3,2‐dioxaborolane (**STBPin)**, (*E*)‐*N*,*N*‐dimethyl‐4‐(4‐(4,4,5,5‐tetramethyl‐1,3,2‐dioxaborolan‐2‐yl)styryl)aniline (**DSTBPin)**, (*E*)‐4‐(4‐(4,4,5,5‐tetramethyl‐1,3,2‐dioxaborolan‐2‐yl)styryl)benzonitrile (**CSTBPin)**, (*E*)‐2‐(4‐(4‐methoxystyryl)phenyl)‐4,4,5,5‐tetramethyl‐1,3,2‐dioxaborolane (**MSTBPin)**, (*E*)‐*N*,*N*‐dimethyl‐4‐(4‐(4,4,5,5‐tetramethyl‐1,3,2‐dioxaborolan‐2‐yl)styryl)naphthalen‐1‐amine (**NDSTBPin**), and *N*,*N*‐dimethyl‐4‐(2‐(4‐(4,4,5,5‐tetramethyl‐1,3,2‐dioxaborolan‐2‐yl)phenyl)oxazol‐5‐yl)aniline (**DAPOX‐BPin**) for the detection of hydrogen peroxide (H_2_O_2_). **DSTBPin** and **MSTBPin** displayed an “Off–On” fluorescence response towards H_2_O_2_, owing to the loss of the intramolecular charge transfer (ICT) excited state. Whereas, **CSTBPin** displayed a decrease in fluorescence intensity in the presence of H_2_O_2_ owing to the introduction of an ICT excited state. **STBPin**, on the other hand, produced a small fluorescence decrease, indicating the importance of an electron‐withdrawing or electron‐donating group in these systems. Unfortunately, the longer wavelength probe, **NDSTBPin**, displayed a decrease in fluorescence intensity. Oxazole‐based probe **DAPOX‐BPin** produced a “turn‐on” response. Regrettably, **DAPOX‐BPin** required large concentrations of H_2_O_2_ (>3 mm) to produce noticeable changes in fluorescence intensity and, therefore, no change in fluorescence was observed in the cell imaging experiments.

Hydrogen peroxide (H_2_O_2_) is the simplest peroxide, playing a significant role as a signaling molecule in a variety of different biological processes.^1^, ^2^ Unfortunately, elevated levels of H_2_O_2_ exceeding the antioxidant capacity results in the damage of multiple cellular components; this is known as “oxidative stress”.^3^ Oxidative stress results in direct or indirect reactive oxygen species (ROS)‐mediated damage to a number of different biological targets such as nucleic acids, lipids, and proteins.^4^, ^5^ Therefore, oxidative stress has been associated with a number of pathological processes including neurodegenerative diseases, diabetes, cancer, and aging.^5^, ^6^, ^7^ For this reason, researchers are actively seeking new and effective approaches for H_2_O_2_ detection. With our research, we are particularly interested in the development of small‐molecule fluorescent probes,^8^, ^9^, ^10^, ^11^, ^12^ as they are well suited to meet the need of tools to map the spatial and temporal distribution of H_2_O_2_ in living cells. However, the major challenge for practical H_2_O_2_ sensing in biological environments is creating water‐soluble systems that respond to H_2_O_2_. Chang and co‐workers^13^, ^14^ as well as others^15^ have developed a range of probes for the selective detection of H_2_O_2_ based on the well‐known hydrogen‐peroxide‐mediated oxidation of arylboronates to phenols. An example of this strategy includes Peroxyfluor‐1 (PF1), a diboronate‐xanthene‐based probe, which is initially non‐fluorescent.^16^ Upon treatment with H_2_O_2_, oxidative deprotection of the boronates results in the generation of the highly fluorescent fluorescein. We decided to utilize this strategy for the development of ratiometric fluorescent probes for the detection of H_2_O_2_. We are particularly interested in ratiometric fluorescence probes, as they provide a method for internal calibration between the reacted and unreacted fluorescent probe. The most commonly exploited systems for the design of ratiometric fluorescent probes are fluorescence resonance energy transfer (FRET) and intramolecular charge transfer (ICT) systems.^17^, ^18^ DiCesare and Lakowicz^19^, ^20^, ^21^ developed a series of ICT‐based stilbene fluorescence probes for the detection of fluoride or saccharides. The addition of each target analyte to these probes resulted in a ratiometric response—a change in emission intensity and wavelength (Scheme 1).

**Scheme 1:**
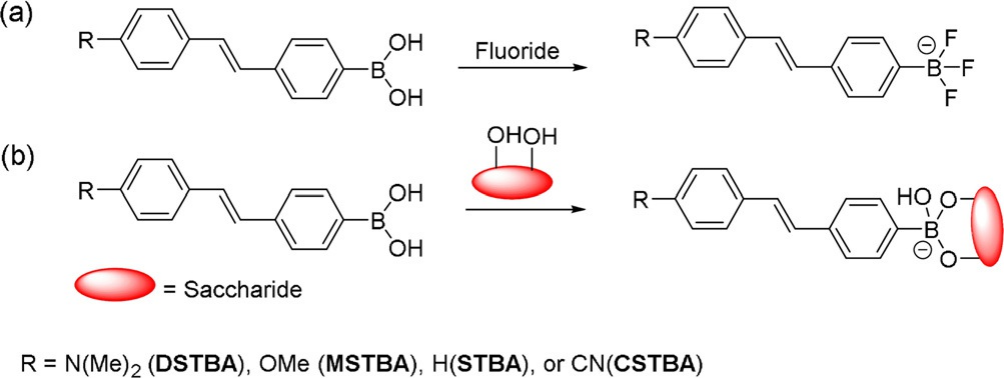
Previous work reported by DiCesare and Lakowicz a) using stilbene boronic acids for the detection of fluoride^20^ and b) using stilbene boronic acids for the detection of saccharides.^21^

Therefore, we chose to evaluate these stilbene‐based fluorescent probes towards H_2_O_2_ detection, as it is believed that the addition of H_2_O_2_ would result in the conversion of the boronate group to a phenol group accompanied by a ratiometric change in fluorescence (Scheme 2). The boronate pinacol ester (BPin) stilbenes were synthesized over the boronic acid (BA) stilbenes, owing to the ease of synthesis and straightforward ^1^H NMR spectroscopic characterization. Note that both BA and BPin would generate the same fluorescence response for H_2_O_2_.

**Scheme 2:**
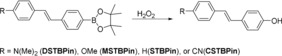
Use of stilbene boronate fluorescent probes for the detection of hydrogen peroxide.

Therefore, we prepared the analogous stilbene boronate ester fluorescent probes (**STBPin**, **DSTBPin**, **MSBPin**, and **CSTBPin**) using a Horner–Wadsworth–Emmons (HWE) reaction to produce a bromo‐substituted intermediate, which was then further reacted with bis(pinacolato)diboron (B_2_pin_2_) using a palladium‐catalyzed Suzuki reaction to produce the pinacol ester probes. All of the synthesized probes contain an sp^2^‐hybridized boronic ester, which is an electron‐withdrawing group (EWG). Therefore, when there is an electron‐donating group (EDG) at the 4′ position, a typical ICT donor–π–acceptor (D‐π‐A) system results, whereas when an EWG is placed at the 4′ position, there is no ICT. Upon the addition of H_2_O_2_, the boronic ester is converted to an electron‐donating phenol, disrupting the charge transfer for these systems.

We initially tested the control probe, **STBPin**, to show that an EDG or EWG was required to produce a change in the fluorescence intensity and emission wavelength. Therefore, the addition of H_2_O_2_ (2 mm) only led to a small decrease in the fluorescence intensity (see Figure S8).

We then evaluated the fluorescence response of **DSTBPin** towards H_2_O_2_ and, as shown in Figure 1, the **DSTBPin** initial emission wavelength was 488 nm. The addition of H_2_O_2_ resulted in a ratiometric response with a blueshift in the emission wavelength from 488 to 444 nm and increase in fluorescence intensity. This observation can be explained through the oxidative conversion of the boronic ester to the electron‐donating phenol, resulting in a change from a Push–Pull ICT mechanism into a Push–Push system, increasing the electron density within the π system.

**Figure 1:**
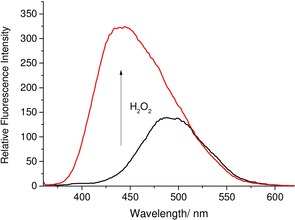
Fluorescence analysis of **DSTBPin** (5 μm) in pH 8.21 buffer solution (52.1 wt % MeOH) with the addition of H_2_O_2_ (2 mm) and re‐analyzed 30 min after H_2_O_2_ addition. *λ*_ex_=350 nm; slit widths: excitation: 10 nm, emission: 3 nm.

The fluorescence characteristics of **MSTBA** were then investigated towards H_2_O_2_. As shown in Figure 2, the emission wavelength shifted from 405 to 380 nm, accompanied by a fluorescence increase upon reaction with H_2_O_2_ (2 mm), similar to **DSTBA**; however, an overall smaller increase in fluorescence intensity was observed. The larger increase in fluorescence intensity for **DSTBA** can be attributed to the dimethlyamino group having a greater electron‐donating ability.

**Figure 2:**
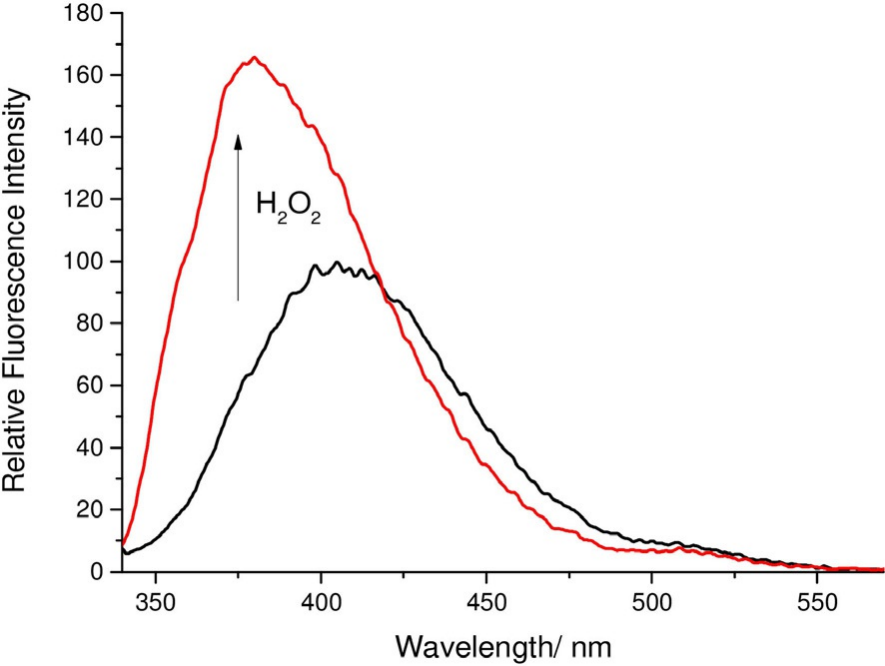
Fluorescence analysis of **MSTBPin** (5 μm) in pH 8.21 buffer solution (52.1 wt % MeOH) with the addition of H_2_O_2_ (2 mm) and re‐analyzed 30 min after H_2_O_2_ addition. *λ*_ex_=330 nm; slit widths: excitation: 10 nm, emission: 3 nm.

The fluorescence characteristics of **CTSBA** were then investigated towards H_2_O_2_. As discussed above, **CTSBA** contains two EWGs, resulting in a Pull–Pull system. The addition of H_2_O_2_ (2 mm) resulted in the introduction of the electron‐donating phenol group, creating a Push–Pull ICT system, accompanied by a decrease in fluorescence intensity (Figure 3).

**Figure 3:**
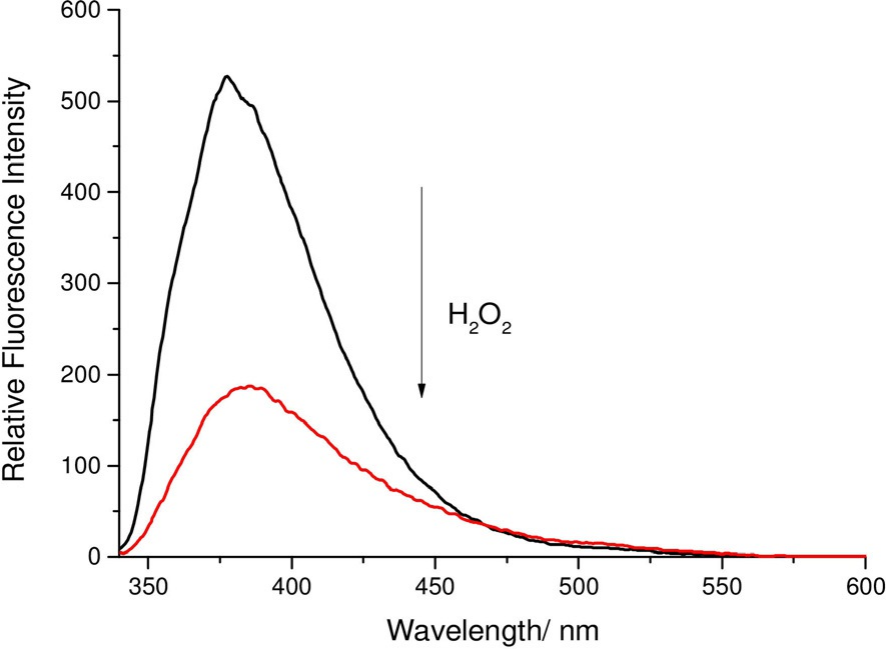
Fluorescence analysis of **CSTBPin** (5 μm) in pH 8.21 buffer solution (52.1 wt % MeOH) with the addition of H_2_O_2_ (2 mm) and re‐analyzed 30 min after H_2_O_2_ addition. *λ*_ex_=330 nm; slit widths: excitation: 10 nm, emission: 3 nm.

Owing to **DSTBPin** demonstrating the biggest “off–on” response upon reaction with H_2_O_2_, we synthesized a naphthalene dimethylamino stilbene boronate (**NDSTBPin**; see Figure 4) fluorescent probe for the detection of H_2_O_2_. This probe was prepared to develop a system with a longer emission wavelength that could overcome the issues associated with background fluorescence of biological materials in cell imaging experiments.

**Figure 4:**
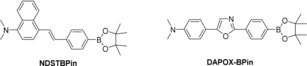
**NDSTBPin** and **DAPOX**‐**BPin** fluorescent probes for the detection of H_2_O_2_.

**NDSTBPin** was synthesized by using the same synthetic procedures as the previously synthesized stilbene fluorescent probes (**STBPin**, **DSTBPin, CSTBPin**, and **MSTBPin**). Dimethylamino‐1‐naphthaldehyde was subjected to a HWE reaction to produce the dimethylamino‐1‐naphthyl bromo‐substituted intermediate, which was then further reacted with B_2_pin_2_ through a palladium‐catalyzed Suzuki reaction to form **NDSTBPin** in a modest yield (33 %).

**NDSTBPin** fluorescence response towards H_2_O_2_ was evaluated. The addition of H_2_O_2_ (2 mm) to **NDSTBPin** resulted in a blueshift in the emission wavelength. Unfortunately, a decrease in fluorescence intensity was observed, unlike **DSTBPin** (Figure 5). This is probably the result of extended conjugation between the BA and the dimethylamino group in **NDSTBPin** when compared to **DSTBPin**, resulting in a fluorescence response similar to **STBPin**. Our main focus was to develop a “turn‐on” H_2_O_2_ fluorescent probe, as it is much easier to visualize a bright signal against a dark background, thus providing easier analysis of H_2_O_2_ in a biological sample.

**Figure 5:**
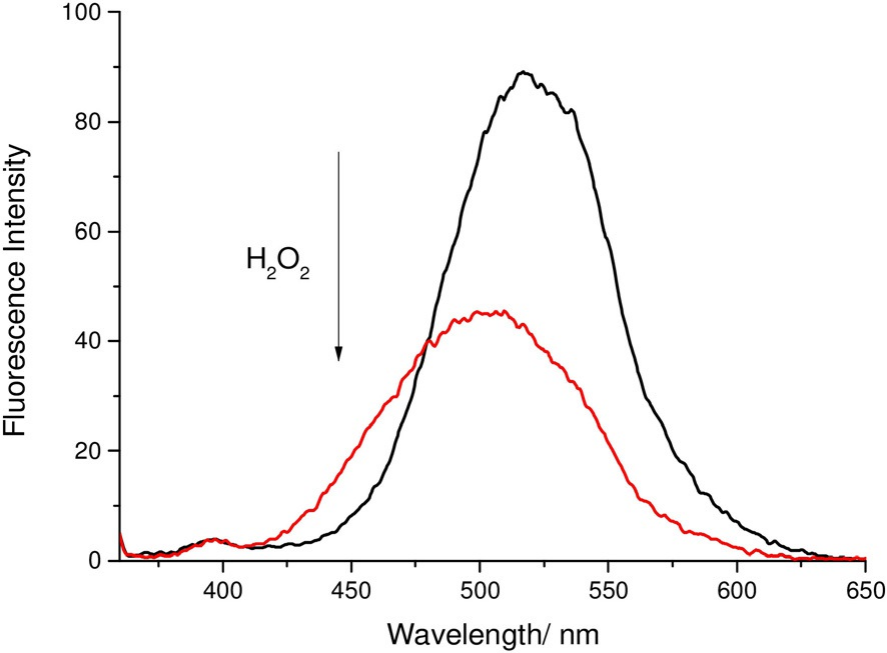
Fluorescence analysis of probe **NDSTBPin** (5 μm) in pH 8.21 buffer solution (52.1 wt % MeOH) with the addition of H_2_O_2_ (2 mm) and re‐analyzed 30 min after H_2_O_2_ addition. *λ*_ex_=350 nm; slit widths: excitation: 10 nm, emission: 3 nm.

Therefore, we turned our attention to the synthesis of an alternative boronate‐based fluorescence probe, a dimethylamino oxazole boronic acid (**DAPOX‐PBA**).^19^, ^20^ **DAPOX‐BPin** was synthesized through the acylation reaction of 2‐amino‐4‐dimethylaminoacetophenone with 4‐bromobenzyl chloride to afford an amide intermediate. This amide intermediate was then dehydrated by using concentrated H_2_SO_4_ to form the desired bromo‐substituted oxazole intermediate. The bromo‐substituted oxazole intermediate was subsequently subjected to a Suzuki–Miyaura reaction, using B_2_pin_2_ to afford **DAPOX‐BPin** in a satisfactory yield (44 %).

As shown in Figure 6, a 3.6‐fold fluorescence increase was observed for **DAPOX‐BPin** with the addition of H_2_O_2_. The favorable fluorescence properties of **DAPOX‐BPin** 20 allowed us to evaluate the detection of H_2_O_2_ with cell imaging experiments. Unfortunately, owing to its poor sensitivity [requiring millimolar concentration with a limit of detection (LOD) greater than 3 mm] towards H_2_O_2_, no change in fluorescence intensity was observed in the cell imaging experiments. Also, no change in fluorescence intensity was observed for the exogenous addition of other ROS/reactive nitrogen species or the endogenous stimulation of ROS (see Figures S9 and S10). This result was unexpected given the relatively low p*K*_a_ (7.8) of **DAPOX‐PBA**.^19^, ^20^ This clearly indicates that a balance must be struck between enhancing the acidity of the BA and maximizing the amount of free sp^2^ boron available for reaction with a nucleophilic oxidant under the measurement conditions (in this case pH=7.25).

**Figure 6:**
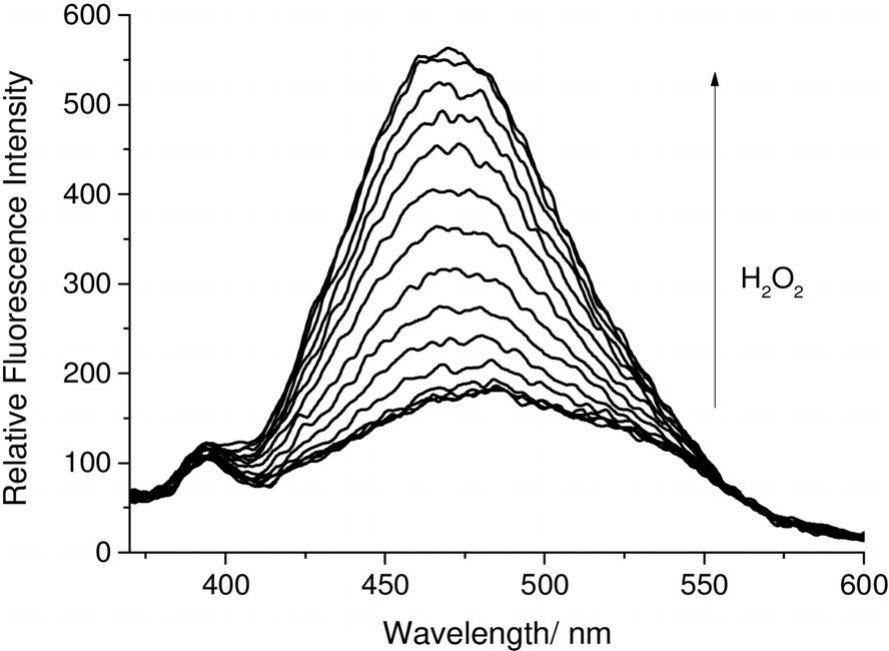
Fluorescent spectra of **DAPOX‐BPin** (30 nm) with the addition of H_2_O_2_ (0 mm −21 mm) in pH 7.25 buffer solution (52.1 wt % MeOH) with a 10 min wait between each measurement. *λ*_ex_=350 nm; slit widths: excitation: 10 nm, emission: 10 nm.

Overall, these boronate stilbene and oxazole fluorescent probes demonstrated a reasonable fluorescence response towards H_2_O_2_. Unfortunately, **DAPOX‐BPin** lacked sensitivity towards H_2_O_2_, requiring non‐biologically relevant concentrations of H_2_O_2_ (millimolar). Therefore, cell imaging experiments using **DAPOX‐BPin** for the detection of H_2_O_2_ resulted in no change of fluorescence intensity. We are currently working to develop a series of ICT fluorescent probes that have enhanced sensitivity towards H_2_O_2_. Our design strategy is to optimize the acidity of the BA group to enhance the reaction with nucleophilic ROS such as H_2_O_2_, whilst maximizing the amount of free sp^2^ boron available for the reaction.

## Experimental Section

All starting materials and reagents were purchased from Sigma Aldrich, Alfa Aesar, Fluorochem, Acros Organics, or Apollo Scientific and used as received without any further purification. Unless otherwise stated, all solvents were of reagent grade and were used without distillation. Dry solvents were obtained from an Innovative Technology Inc. PS‐400‐7 solvent purification system. All water used was distilled. Stock solutions of H_2_O_2_ were prepared from commercially available (Sigma Aldrich) hydrogen peroxide (30 % in H_2_O) and diluted accordingly. Phosphate buffer solution (52.1 wt % MeOH) was prepared according to the literature.^22^ Thin‐layer chromatography was performed by using commercially available Macherey–Nagel aluminum‐backed plates coated with a 0.20 mm layer of silica gel (60 Å) containing fluorescent indicator UV254. These plates were visualized by using either ultraviolet light with a wavelength of 254 or 365 nm, or by staining the plates with vanillin or ninhydrin solution. Silica gel column chromatography was carried out by using Fisher or Sigma Aldrich 60 Å silica gel (35–70 μm).

Unless otherwise stated, all NMR spectra were obtained by using a Bruker Advance 300, with all spectra recorded in chloroform‐*d* or [*D*_6_]DMSO. ^1^H NMR spectra were recorded at an operating frequency of 300 MHz, ^11^B NMR spectra were recorded at an operating frequency of 96 MHz, and ^13^C NMR spectra were recorded at an operating frequency of 75 MHz, with proton decoupling for all ^13^C NMR spectra. High‐resolution mass spectrometry (HRMS) results were typically acquired on an externally calibrated Bruker Daltonics micrOTOF time‐of‐flight mass spectrometer coupled to an electrospray source (ESI‐TOF). Fluorescence measurements were performed on a PerkinElmer luminescence spectrophotometer LS 50B/ LS 55 B utilizing a Starna silica (quartz) cuvette with a 10 mm path length (four faces polished). Data were collected by using the PerkinElmer FL Winlab software package. All solvents used in the fluorescence measurements were HPLC or fluorescence grade and the water was deionized. Further reprocessing of the data was carried in OriginPro 8.0 software. All pH measurements taken during fluorescence/absorption experiments were recorded on a Hanna Instruments HI 9321 microprocessor pH meter, which was routinely calibrated by using Fisher Chemicals standard buffer solutions (pH 4.0: phthalate; 7.0: phosphate; 10.0: borate). UV/Vis measurements were performed on a PerkinElmer Lambda 20 Spectrophotometer, utilizing a Starna silica (quartz) cuvette with a 10 mm path lengths (two faces polished). Data were collected by using the PerkinElmer UVWinlab software package. Further reprocessing of the data was carried in OriginPro 8.0 software.

See the Supporting Information for full synthetic experimental procedures.

## Conflict of interest

*The authors declare no conflict of interest*.

## Supporting information

As a service to our authors and readers, this journal provides supporting information supplied by the authors. Such materials are peer reviewed and may be re‐organized for online delivery, but are not copy‐edited or typeset. Technical support issues arising from supporting information (other than missing files) should be addressed to the authors.

Supplementary
