# Supplementary material for: Boronate‐Based Fluorescence Probes for the Detection of Hydrogen Peroxide
Source: ChemistryOpen. 2018 Jan 26;7(3):262–5. doi: 10.1002/open.201700189 (PMC5838387; doi:10.1002/open.201700189)
Supplement: Supplementary file 1 — Supplementary [file OPEN-7-262-s001.pdf]

ChemistryOPEN

# Supporting Information

© 2018 The Authors. Published by Wiley-VCH Verlag GmbH & Co. KGaA, Weinheim

# Boronate-Based Fluorescence Probes for the Detection of Hydrogen Peroxide

Emma V. Lampard,[a] Adam C. Sedgwick,*[a] Xiaolong Sun,[b] Katherine L. Filer,[a] Samantha C. Hewins,[a] Gyoungmi Kim,[c] Juyoung Yoon,[c] Steven D. Bull,[a] and Tony D. James*[a]

open_201700189_sm_miscellaneous_information.pdf

Supporting Information

**Content**


1. **UV and Fluorescence analysis**
2. **Cell imaging**
3. **Experimental**
4. **NMR**
5. **References**

## 1. UV and Fluorescence analysis

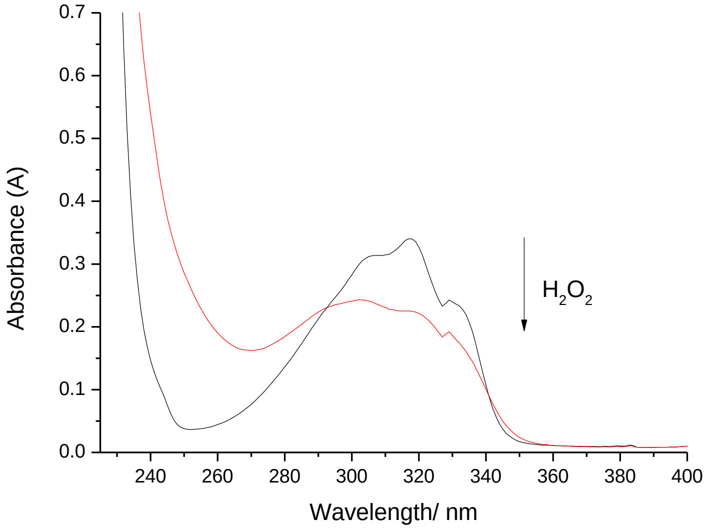


**Figure S1** - UV analysis of **STBPin** (10 µM) in pH 8.21 buffer (52.1 wt% MeOH) with (**red**) and without (**black**) the presence of $H_2O_2$ (2 millimolar). 1 hr between measurement

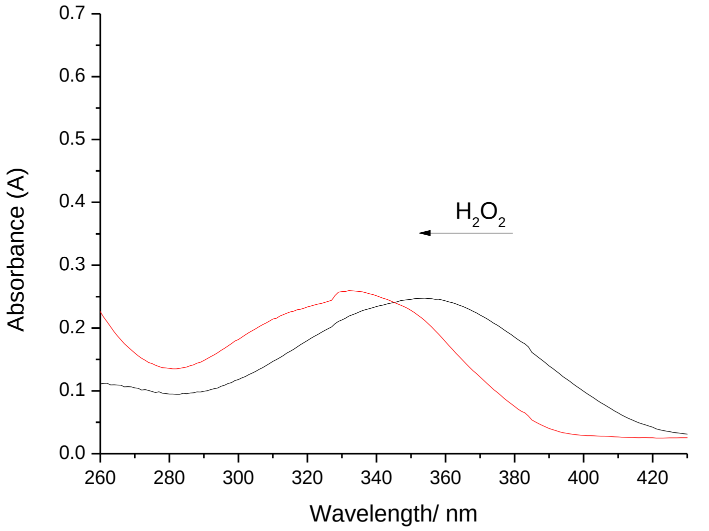


**Figure S2** - UV analysis of **DSTBPin** (10 µM) in pH 8.21 buffer (52.1 wt% MeOH) with (**red**) and without (**black**) the presence of $H_2O_2$ (2 millimolar). 1 hr between measurement

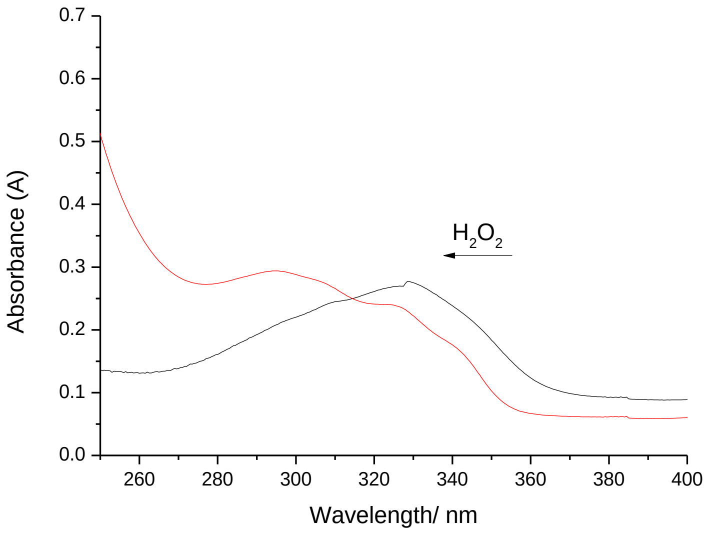


**Figure S3** - UV analysis of **MSTBPin** (10 µM) in pH 8.21 buffer (52.1 wt% MeOH) with (**red**) and without (**black**) the presence of $H_2O_2$ (2 millimolar). 1 hr between measurement

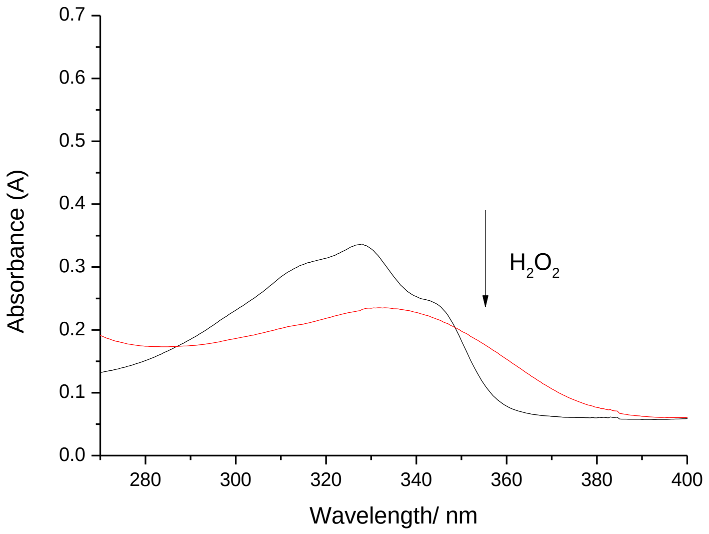


**Figure S4** - UV analysis of **CSTBPin** (10 µM) in pH 8.21 buffer (52.1 wt% MeOH) with (**red**) and without (**black**) the presence of $H_2O_2$ (2 millimolar). 1 hr between measurement

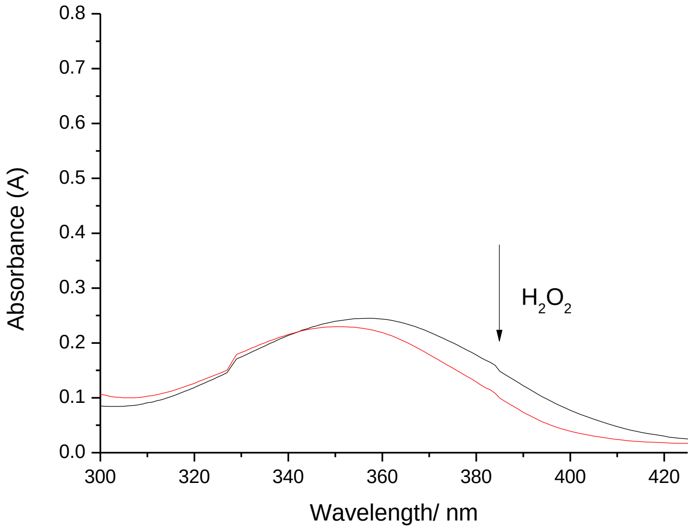


**Figure S5** - UV analysis of **NDSTBPin** (10 µM) in pH 8.21 buffer (52.1 wt% MeOH) with (**red**) and without (**black**) the presence of $H_2O_2$ (2 millimolar). 1 hr between measurement

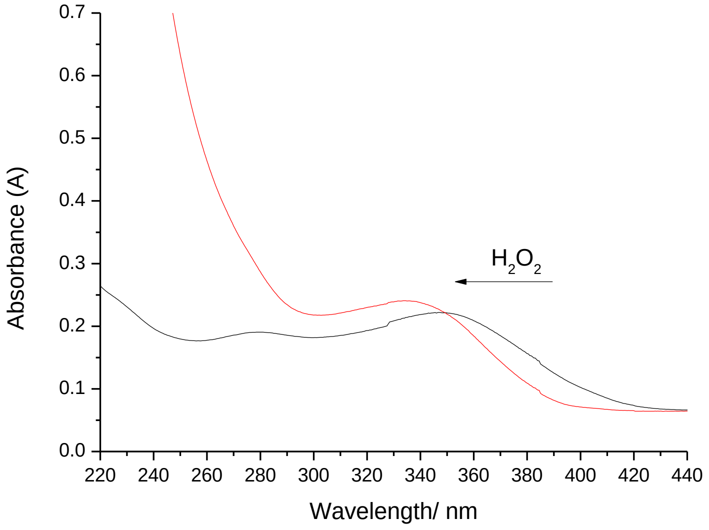


**Figure S6** - UV analysis of **DAPOX-BPin** (10 µM) in pH 8.21 buffer (52.1 wt% MeOH) with (**red**) and without (**black**) the presence of $H_2O_2$ (2 millimolar). 1 hr between measurement

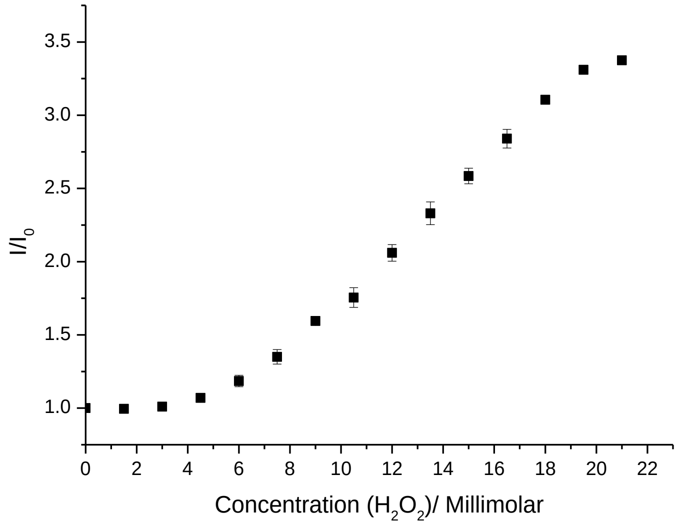


**Figure S7** - Fluorescence intensity changes (I/Io) for **DAPOX-BPin** (30 nM) with the addition of $H_2O_2$ (0 – 21 Mm) in pH 8.21 buffer (52.1 wt% MeOH). $\lambda_{ex}$ = 350 nm/$\lambda_{em}$ = 455 nm. Slit widths ex = 10 nm and em = 10 nm. 15 min wait between each measurement.

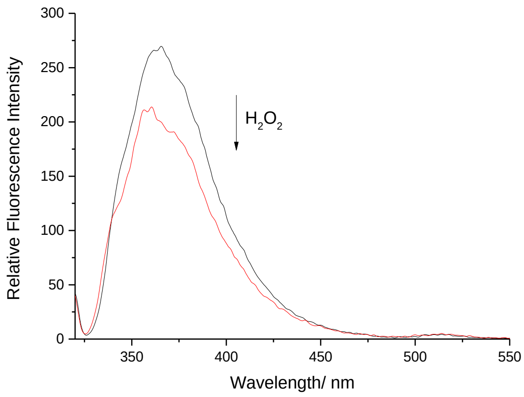


**Figure S8** - Fluorescence analysis of probe **STBPin** (5 µM) in pH 8.21 buffer solution (52.1 wt% MeOH) with the addition of $H_2O_2$ (2 mM) and re-analyzed 30 minutes after $H_2O_2$ addition. $\lambda_{ex}$ = 315 nm; slit widths: excitation: 10 nm, emission: 3 nm.

**Table S1 -** A summary of the fluorescence analysis data for the boronate probes

| Probe | Fluorescence Change upon addition of $H_2O_2$ |
|---|---|
| **STBPin** | Small intensity decrease |
| **DSTBPin** | Blue $\lambda_{em}$ shift, intensity increase |
| **MSTBPin** | Blue $\lambda_{em}$ shift, intensity increase |
| **CSTBPin** | Large intensity decrease |
| **NDSTBPin** | Blue $\lambda_{em}$ shift, intensity decrease |
| **DAPOX-BPin** | Blue $\lambda_{em}$ shift, intensity increase |

## 2. Cell imaging

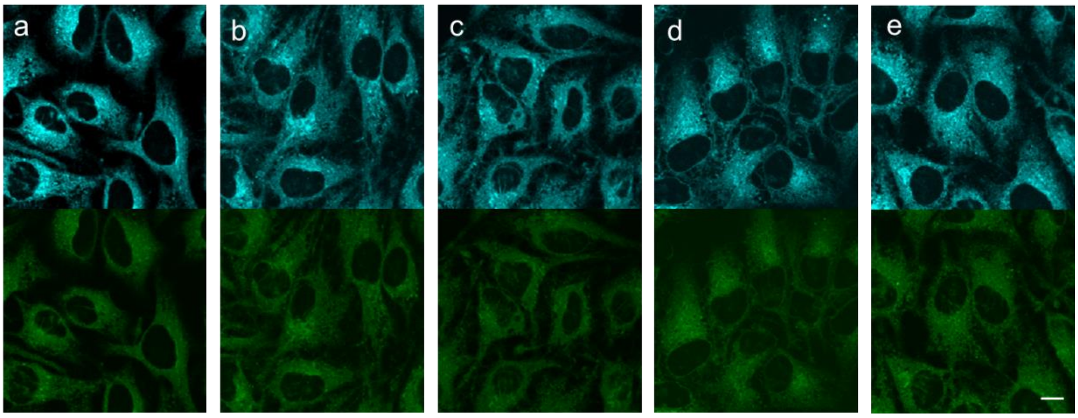


**Figure S9** - HeLa cells were incubated with **DAPOX-BPin (**5 μM probe) for 15 min and washed with DPBS and treated with (a) no, (b) 300 μM $H_2O_2$, (c) 300 μM $KO_2$, (d) 300 μM NaOCl, (e) 300 μM $ONOO^-$ for 15 min and acquired fluorescence images by confocal microscopy. Cyan: ex. 405nm/em. 465-495nm, green: ex. 405nm/em. 490-590nm. Scale bar: 10 μm.

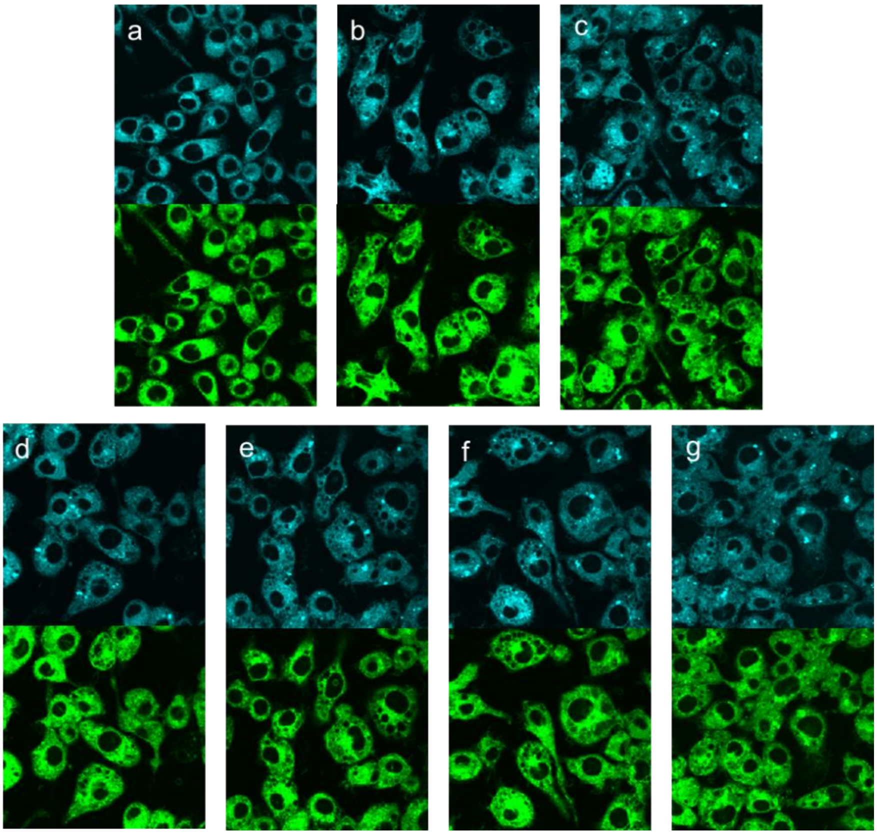


**Figure S10 -** RAW 264.7 cells were incubated with 500 ng/ml LPS, 50 ng/ml IFN-γ for 4 hr and washed with DPBS and treated with (a) no, (b-g all) LPS, IFN-γ, (c) DMTU ($H_2O_2$ scavenger), (d) ebselen ($ONOO^-$ scavenger), (e) mannitol (·OH scavenger), (f) $NaN_3$ (singlet oxygen scavenger), (g) tiron (superoxide scavenger) and stained with **DAPOX-BPin** (5 Mm) for 15min. After washing with DPBS, the fluorescence images were acquired by confocal microscopy.

## 3. Experimental

**General procedures:**

**General Procedure 1 – Horner-Wadsworth-Emmons Reaction for Synthesis of Bromo-Substituted Intermediates**

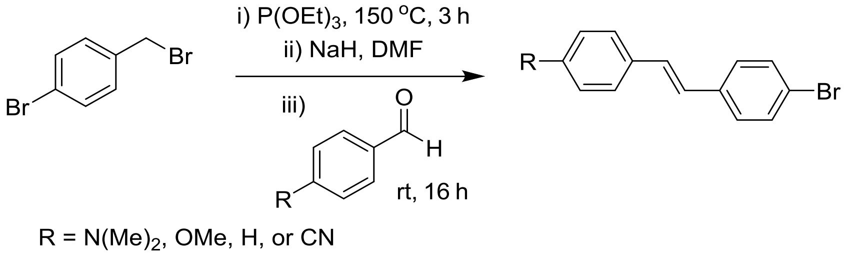


4-Bromobenzyl bromide (1.0 equiv.) and triethyl phosphite (1.2 equiv.) were heated to 150 °C with stirring for 3 hours under $N_2$. Reaction completion was confirmed by the disappearance of the P-OEt peak at δ 139.69 ppm and the appearance of the P=O peak at δ 26.34 ppm in the $^{31}P$ NMR spectrum. The reaction mixture was cooled to room temperature, and anhydrous DMF (15 mL) was added. The resulting solution was cooled in an ice bath and NaH – 60 % in mineral oil (1.5 equiv.) was added. After stirring at 0 °C for 20 minutes, a solution of the corresponding aldehyde (1.0 equiv.) in anhydrous DMF (5 mL) was slowly added. The resulting slurry was allowed to warm to room temperature and left to stir overnight. The reaction mixture was poured into ice water and left to stir until the ice had melted. The precipitated product was collected by filtration, washed with water and dried under vacuum.

**General Procedure 2 – Suzuki Reaction for Synthesis of Boronic Acid Pinacol Ester Probes**

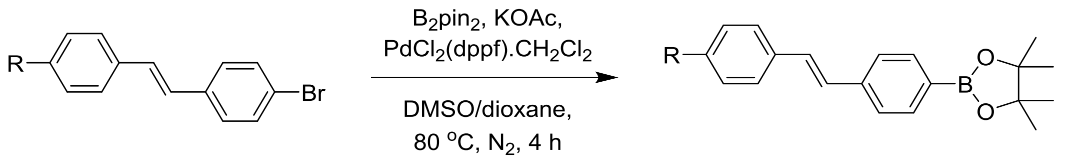


R = $N(Me)_2$, OMe, H, or CN

The corresponding bromo-substituted styrene intermediate (1.0 equiv.), bis(pinacolato)diboron (1.07 equiv.) and KOAc (2.91 equiv.) were partially dissolved in DMSO/dioxane (6 mL/2 mL). $PdCl_2(dppf).CH_2Cl_2$ (0.04 equiv.) was partially dissolved in DMSO/dioxane (3 mL/1 mL) and was added drop-wise to the reaction mixture. The reaction was heated to 80 °C and stirred under $N_2$ for 4 hours. The reaction mixture was cooled to room temperature and quenched with saturated $NH_4Cl$. The crude product was extracted 3 times into ethyl acetate. The combined organic layers were dried over MgSO4, filtered, and the solvent was removed under reduced pressure to give the crude reaction product.

**Synthesis of STBPin, DSTBPin, MSTBPin and CSTBPin**

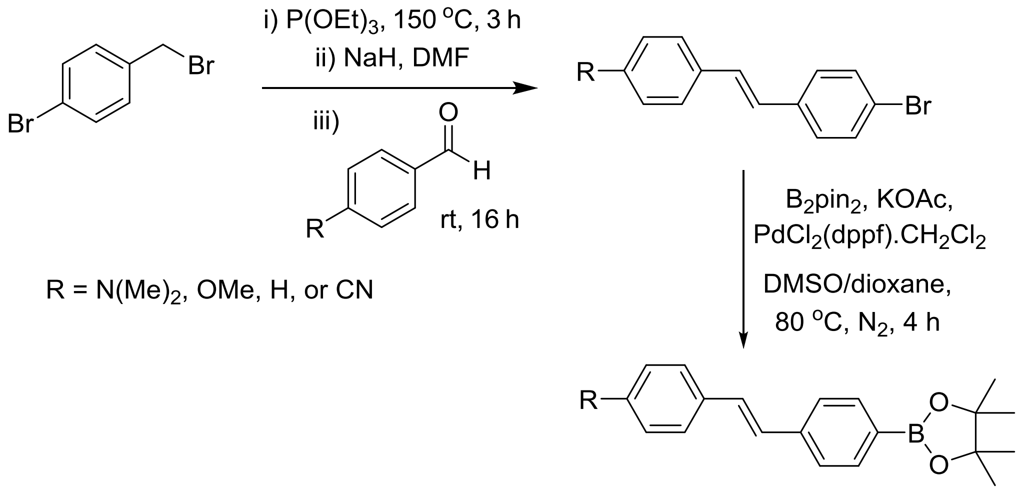


**Scheme S1** – Synthesis of **STBA**, **DSTBA**, **MSTBA** and **CSTBA**

**(*E*)-4-(4-bromostyryl)-*N*,*N*-dimethylaniline (1)**

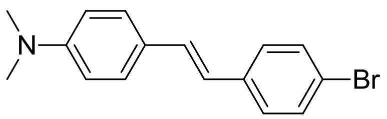

The title compound was prepared according to General Procedure 1 using 4-bromobenzyl bromide (1.68 g, 6.71 mmol), triethyl phosphite (1.40 mL, 8.05 mmol), NaH – 60 % in mineral oil (0.40 g, 10.07 mmol) and 4-(dimethylamino)benzaldehyde (1.00 g, 6.71 mmol). The crude reaction product was recrystallized in DCM/hexane. The purified product was collected as a pale yellow crystalline solid in 73 % yield. $^{1}$H NMR (300 MHz, $CDCl_3$): δ 7.49 - 7.29 (6H, ArH, m), 7.03 (1H, alkene CH, d, J = 16.3 Hz), 6.83 (1H, alkene CH, d, J = 16.3 Hz), 6.71 (2H, ArH, d, J = 8.7 Hz), 2.99 (6H, $CH_3$, s); $^{13}$C NMR (75.5 MHz, $CDCl_3$): δ 150.27, 137.17, 131.64, 129.54, 127.69, 127.48, 125.26, 122.96, 120.13, 112.39, 40.46; FTIR ν ($cm^{-1}$): 2796, 1065, 967, 819; HRMS (ES) m/z calculated for $C_{16}H_{17}NBr$: $[M+H]^+$ 302.0544, found 302.0526; M.p.: 207-209 °C.

**(*E*)-1-bromo-4-(4-methoxystyryl)benzene (2)**

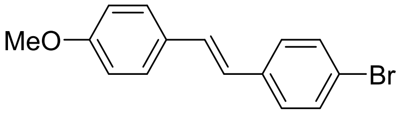


The title compound was prepared according to General Procedure 1 using 4-bromobenzyl bromide (1.68 g, 6.71 mmol), triethyl phosphite (1.40 mL, 8.05 mmol), sodium hydride (0.40 g, 10.07 mmol) and 4-methoxybenzaldehyde (0.81 mL, 6.71 mmol). The crude reaction product was recrystallized in DCM/hexane. The purified product was collected as a white crystalline solid in 60 % yield. $^1$H NMR (300 MHz, $CDCl_3$): δ 7.50 – 7.40 (4H, ArH, m), 7.39 – 7.31 (2H, ArH, m), 7.05 (1H, alkene CH, d, J = 16.3 Hz), 6.90 (1H, alkene CH, d, J = 16.3 Hz), 6.93 – 6.86 (2H, ArH, m), 3.83 (3H, $OCH_3$). All data matches literature data.[1]

**(*E*)-1-bromo-4-styrylbenzene (3)**

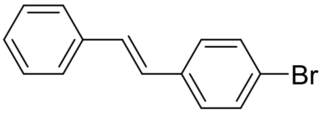


The title compound was prepared according to General Procedure 1 using 4-bromobenzyl bromide (1.68 g, 6.71 mmol), triethyl phosphite (1.40 mL, 8.05 mmol), sodium hydride (0.40 g, 10.07 mmol) and benzaldehyde (0.68 mL, 6.71 mmol). The crude reaction product was recrystallized in DCM/hexane. The purified product was collected as a white crystalline solid in 47 % yield. $^1$H NMR (300 MHz, $CDCl_3$): δ 7.55-7.44 (4H, ArH, m), 7.42-7.33 (4H, ArH, m), 7.32-7.24 (1H, ArH, m), 7.11 (1H, alkene CH, d, J = 16.3 Hz), 7.03 (1H, alkene CH, d, J = 16.3 Hz); 13C NMR (75.5 MHz, $CDCl_3$): δ 136.97, 136.30, 131.80, 129.45, 128.77, 128.00, 127.93, 127.42, 126.59, 121.33; FTIR ν ($cm^{-1}$): 2925, 1484, 1003, 811; M.p.: 134-136 ℃. All data matches the literature reported data [1]

**(*E*)-4-(4-bromostyryl)benzonitrile (4)**

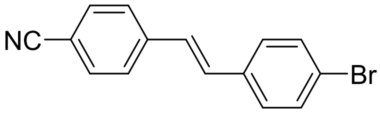


The title compound was prepared according to General Procedure 1 using 4-bromobenzyl bromide (1.68 g, 6.71 mmol), triethyl phosphite (1.40 mL, 8.05 mmol), sodium hydride (0.40 g, 10.07 mmol) and 4-cyanobenzaldehyde (0.88 g, 6.71 mmol). The crude reaction product was recrystallized in DCM/hexane. The purified product was collected as a yellow crystalline solid in 29 % yield. $^{1}H$ NMR (300 MHz, $CDCl_3$): δ 7.67-7.54 (4H, ArH, m), 7.54-7.47 (2H, ArH, m), 7.14 (2H, ArH, m), 7.14 (1H, alkene CH, d, J = 16.3 Hz), 7.06 (1H, alkene CH, d, J = 16.3 Hz); 13C NMR (75.5 MHz, $CDCl_3$): δ 141.44, 135.23, 132.58, 132.03, 131.09, 128.37, 127.41, 126.95, 122.55, 119.00, 110.87; FTIR ν ($cm^{-1}$): 2975, 2219, 1599, 961, 825; HRMS (ES) m/z calculated for $C_{15}H_{10}NBrNa$: $[M+Na]^+$ 305.9894, found 305.9877; Mp: 190-192 ℃

**(*E*)-*N*,*N*-dimethyl-4-(4-(4,4,5,5-tetramethyl-1,3,2-dioxaborolan-2-yl)styryl)aniline (5)**

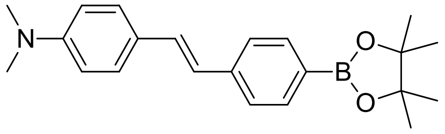

The title compound was prepared according to General Procedure 2 using (E)-4-(4-bromostyryl)-N,N-dimethylaniline (0.50 g, 1.66 mmol), bis(pinacolato)diboron (0.45 g, 1.78 mmol), KOAc (0.47 g, 4.83 mmol) and $PdCl_2$(dppf).$CH_2Cl_2$ (50 mg, 0.06 mmol). The crude reaction product was recrystallized in DCM/hexane. The purified product was collected as a yellow fibrous solid in 52% yield. $^1$H NMR (300 MHz, $CDCl_3$): δ 7.77 (2 H, ArH, d, J = 8.2 Hz), 7.50 – 7.37 (4 H, ArH, m), 7.12 (1 H, alkene CH, d, J = 16.3 Hz), 6.91 (1 H, alkene CH, d, J = 16.3 Hz), 6.71 ( 2 H, ArH, d, J = 8.9 Hz), 2.99 (6 H, $CH_3$, s), 1.35 (12 H, pinacol ester, $CH_3$, s); $^{13}$C NMR (75.5 MHz CDCl3): δ 150.23, 140.98, 135.12, 129.82, 127.76, 125.54, 125.31, 124.21, 112.40, 83.71, 40.48, 24.91; $^{11}$B NMR (96 MHz, CDCl3): δ 32.64; FTIR ν ($cm^{-1}$): 2796, 1596, 1354, 966; HRMS (ES) m/z calculated for $C_{22}H_{28}NO_2BNa$: $[M+Na]^+$ 372.2111, found 372.2127; Mp: 184-187 ℃

**(*E*)-2-(4-(4-methoxystyryl)phenyl)-4,4,5,5-tetramethyl-1,3,2-dioxaborolane (6)**

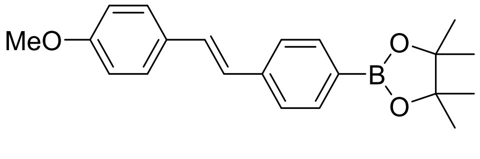

The title compound was prepared according to General Procedure 2 using (E)-1-bromo-4-(4-methoxystyryl)benzene (0.48 g, 1.66 mmol), bis(pinacolato)diboron (0.45 g, 1.78 mmol), KOAc (0.47 g, 4.83 mmol) and $PdCl_2(dppf).CH_2Cl_2$ (50 mg, 0.06 mmol). The crude reaction product was recrystallized in DCM/hexane. The purified product was collected as a pale brown solid in 36 % yield. $^{1}$H NMR (300 MHz, $CDCl_3$): δ 7.79 (2 H, ArH, d, J = 8.1 Hz), 7.54-7.43 (4 H, ArH, m), 7.14 (1 H, alkene CH, d, J = 16.3 Hz), 6.98 (1 H, alkene CH, d, J = 16.3 Hz), 6.90, (2 H, ArH, d, J = 8.8 Hz), 3.83 (3 H, $OCH_3$, s), 1.35 (12 H, pinacol ester $CH_3$, s); $^{13}$C NMR (75.5 MHz, $CDCl_3$): δ 159.46, 140.39, 135.15, 130.02, 129.19, 127.87, 126.54, 125.55, 114.17, 83.76, 55.34, 24.90; $^{11}$B NMR (96 MHz, $CDCl_3$): δ 33.28; FTIR ν ($cm^{-1}$): 2974, 1599, 1357, 963, 830; HRMS (ES) m/z calculated for $C_{21}H_{25}O_3BNa$: $[M+Na]^+$ 359.1794, found 359.1775; Mp: 171-173 ℃

**(*E*)-4,4,5,5-tetramethyl-2-(4-styrylphenyl)-1,3,2-dioxaborolane (7)**

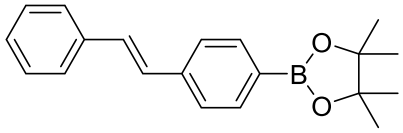

The title compound was prepared according to General Procedure 2 using (E)-1-bromo-4-styrylbenzene (0.43 g, 1.66 mmol), bis(pinacolato)diboron (0.45 g, 1.78 mmol), KOAc (0.47 g, 4.83 mmol) and $PdCl_2$(dppf).$CH_2Cl_2$ (50 mg, 0.06 mmol). The crude reaction product was recrystallized in hot MeOH. The purified product was collected as a pale brown solid in 47 % yield. $^1$H NMR (250 MHz, $CDCl_3$): δ 7.84 (2 H, ArH, d, J = 7.7 Hz), 7.56 (4 H, ArH, d, J = 7.6 Hz), 7.40 (2 H, ArH, t, J = 7.4 Hz), 7.35-7.29 (1 H, ArH, m), 7.23 (1 H, alkene CH, d, J = 16.3 Hz), 7.14 (1 H, alkene CH, d, J = 16.3 Hz), 1.39 (12 H, pinacol ester $CH_3$, s); $^{13}$C NMR (75 MHz, $CDCl_3$): δ 140.03, 137.20, 135.18, 129.66, 128.72, 128.64, 127.82, 126.64, 125.82, 83.81, 24.90; $^{11}$B NMR (96 MHz, $CDCl_3$): δ 33.81; FTIR ν (cm-1): 2979, 1604, 1357, 1140, 963; Mp: 125-127 °C. All data matches the literature reported data [2]

**(*E*)-4-(4-(4,4,5,5-tetramethyl-1,3,2-dioxaborolan-2-yl)styryl)benzonitrile (8)**

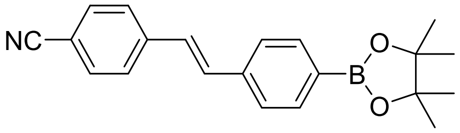


The title compound was prepared according to General Procedure 2 using (E)-4-(4-bromostyryl)benzonitrile (0.47 g, 1.66 mmol), bis(pinacolato)diboron (0.45 g, 1.78 mmol), KOAc (0.47 g, 4.83 mmol) and $PdCl_2$(dppf).$CH_2Cl_2$ (50 mg, 0.06 mmol). The crude reaction product was recrystallized in DCM/hexane. The purified product was collected as a pale brown solid in 60 % yield. $^{1}H$ NMR (300 MHz, $CDCl_3$): δ 7.82 (2 H, ArH, d, J = 8.1 Hz), 7.61 (4 H, ArH, q, J = 8.6 Hz), 7.53 (2 H, ArH, d, J = 8.1 Hz), 7.23 (1 H, alkene CH, d, J = 16.3 Hz), 7.14 (1 H, alkene CH, d, J = 16.3 Hz), 1.36 (12 H, pinacol ester $CH_3$, s); $^{13}C$ NMR (75.5 MHz, $CDCl_3$): δ 141.71, 138.87, 135.28, 132.52, 132.34, 127.62, 126.98, 126.19, 119.02, 110.76, 83.94, 24.90; $^{11}B$ NMR (96 MHz, $CDCl_3$): δ 35.53; FTIR ν ($cm^{-1}$): 2977, 2221, 1354, 959, 653; HRMS (ES) m/z calculated for $C_{21}H_{22}NBO_2Na$: $[M+Na]^+$ 354.1641, found 354.1617; Mp: 192-194 °C

## Synthesis of NDSTBPin

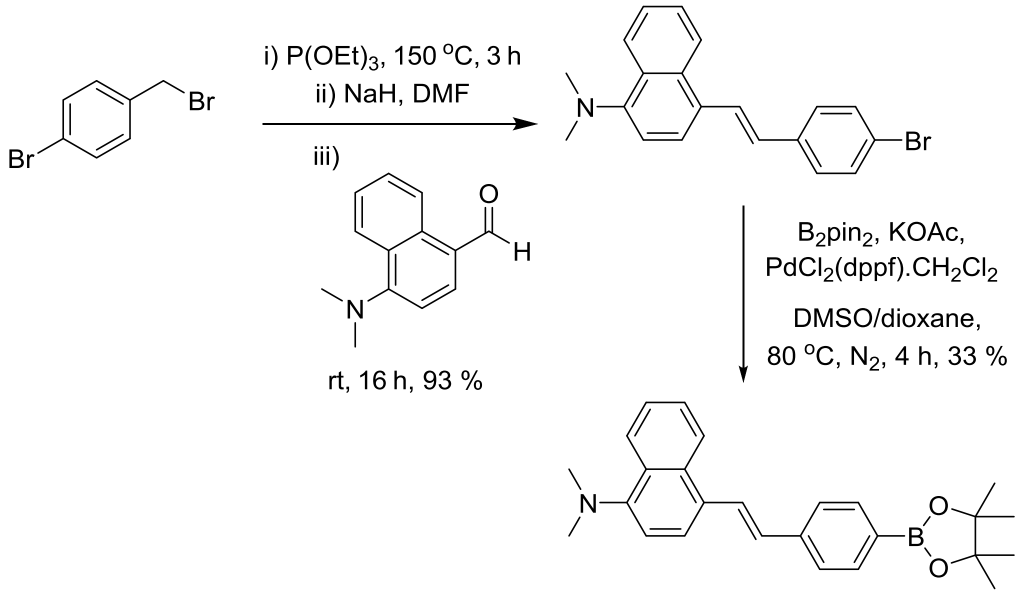


**Scheme S2** – Synthesis of **NDSTBA**

**(E)-4-(4-bromostyryl)-N,N-dimethylnaphthalen-1-amine (9)**

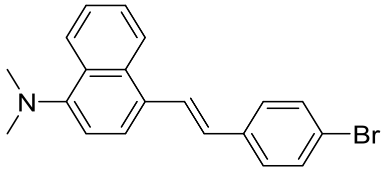


The title compound was prepared according to General Procedure 1 using 4-bromobenzyl bromide (0.84 g, 3.36 mmol), triethyl phosphite (0.70 mL, 4.03 mmol), NaH – 60 % in mineral oil (0.20 g, 5.04 mmol) and 4-dimethylamino-1-naphthaldehyde (0.67 g, 3.36 mmol). The precipitated product was collected by filtration, washed with water and dried under vacuum to give a yellow solid in 93 % yield. $^{1}$H NMR (250 MHz, $CDCl_3$): δ 8.35-8.24 (1 H, ArH, m), 8.22-8.12 (1 H, ArH, m), 7.83 (1 H, alkene CH, d, J = 16.0 Hz), 7.67 (1 H, ArH, d, J = 7.9 Hz), 7.59-7.38 (6 H, ArH, m), 7.09 (1 H, ArH, d, J = 7.9 Hz), 7.00 (1 H, alkene CH, d, J = 16.0 Hz), 2.93 (6H, $CH_3$, s); $^{13}$C NMR (75.5 MHz, $CDCl_3$): δ 151.21, 136.89, 132.54, 131.81, 129.27, 128.86, 128.69, 128.01, 126.68, 126.10, 125.17, 124.84, 124.09, 123.86, 121.09, 113.87, 45.21; FTIR ν ($cm^{-1}$): 2925, 2785, 961, 825; HRMS (ES) m/z calculated for $C_{20}H_{19}NBr$: $[M+H]^+$ 352.0701, found 352.0675; Mp: 92-97 °C

**(*E*)-*N*,*N*-dimethyl-4-(4-(4,4,5,5-tetramethyl-1,3,2-dioxaborolan-2-yl)styryl)naphthalen-1-amine (10)**

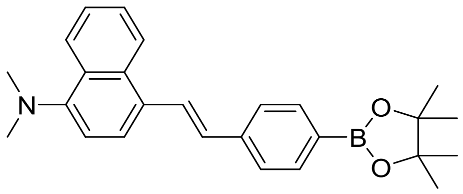


The title compound was prepared according to General Procedure 2 using (E)-4-(4-bromostyryl)-N,N-dimethylnaphthalen-1-amine (0.58 g, 1.66 mmol), bis(pinacolato)diboron (0.45 g, 1.78 mmol), KOAc (0.47 g, 4.83 mmol) and $PdCl_2$(dppf).$CH_2Cl_2$ (50 mg, 0.06 mmol). The reaction was heated to 80 ℃ and stirred under $N_2$ for an extended reaction time of 18 hrs. The crude reaction product was recrystallized in hot MeOH. The purified product was collected as a dark green solid in 33 % yield. $^1$H NMR (300 MHz, $CDCl_3$): δ 8.31-8.25 (1 H, ArH, m), 8.24-8.18 (1 H, ArH, m), 7.91 (1 H, alkene CH, d, J = 16.0 Hz), 7.83 (2 H, ArH, d, J = 8.0 Hz), 7.69 (1 H, ArH, d, J = 7.9 Hz), 7.59 (2 H, ArH, d, J = 8.0 Hz), 7.56-7.48 (2 H, ArH, m), 7.11 (1 H, ArH, d, J = 7.9 Hz), 7.07 (1 H, alkene CH, d, J = 16.0 Hz), 2.92 (6 H, $CH_3$, s), 1.37 (12 H, pinacol ester $CH_3$, s); 13C NMR (75 MHz, CDCl3): δ 151.11, 140.64, 135.22, 132.62, 130.07, 129.54, 128.74, 126.87, 126.04, 125.81, 125.12, 124.77, 124.20, 123.85, 113.89, 83.81, 45.21, 24.92; 11B NMR (96 MHz, CDCl3): δ 35.94; FTIR ν (cm-1): 2979, 2829, 1604, 1357, 1140, 760; HRMS (ES) m/z calculated for C26H31N1B1O2: [M+H]+ 400.2448, found 400.2427; Mp: 159-164 ℃

## Synthesis of DAPOX-BPin

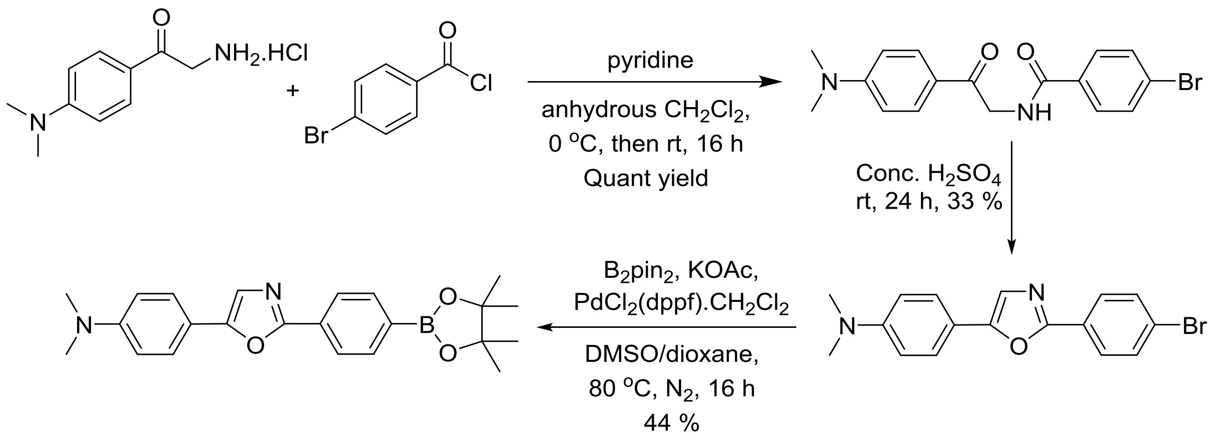


**Scheme S3 -** Synthesis of **DAPOX-BPin**

### ***N,N*-dimethyl-4-(2-(4-(4,4,5,5-tetramethyl-1,3,2-dioxaborolan-2-yl)phenyl)oxazol-5-yl)aniline (11)**

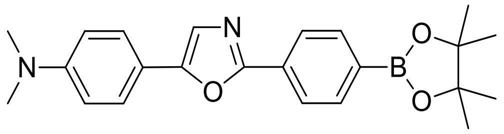

2-Amino-4-dimethylaminoacetophenone hydrochloride (100 mg, 0.47 mmol) and 4-bromobenzoyl chloride (204 mg, 0.94 mmol) were suspended in anhydrous DCM (40 mL) and the resulting mixture was cooled down to 0 °C. Anhydrous pyridine (188 µL) was added drop-wise to the reaction mixture with stirring. The resulting mixture was allowed to warm to room temperature and left to stir overnight. The reaction mixture was diluted with $CHCl_3$, and the resulting solution was washed twice with water. The organic layer was dried over $MgSO_4$, filtered, and the solvent was removed under reduced pressure to give a pale brown powder in

quantitative yield. 4-bromo-N-(2-(4-(dimethylamino)phenyl)-2-oxoethyl)benzamide (200 mg, 0.55 mmol) was then suspended in concentrated $H_2SO_4$ (1 mL) and stirred for 24 hrs at rt. The resulting solution was poured onto ice. The precipitated product was collected by filtration, washed with water and dried under vacuum. The crude product was dissolved in DCM and washed twice with 1 M NaOH to remove any unreacted 4-bromobenzoyl chloride leftover from the previous reaction. The organic layer was dried over $MgSO_4$, filtered, and the solvent was removed under reduced pressure to give a pale yellow powdery solid in 34% yield. 4-(2-(4-bromophenyl)oxazol-5-yl)-N,N-dimethylaniline (47 mg, 0.14 mmol), bis(pinacolato)diboron (38 mg, 0.15 mmol) and KOAc (40 mg, 0.41 mmol) were partially dissolved in 3:1 DMSO/dioxane (2 mL). $PdCl_2$(dppf).$CH_2Cl_2$ (4.6 mg, 0.006 mmol) was partially dissolved in 3:1 DMSO/dioxane (0.75 mL) and was added drop-wise to the reaction mixture. The reaction was heated to 80 ℃ and stirred under N2 for 16 hours. The reaction mixture was cooled to room temperature and quenched with saturated $NH_4Cl$. The crude product was extracted 3 times into EtOAc. The combined organic layers were dried over $MgSO_4$, filtered, and the solvent was removed under reduced pressure to give a yellowy green solid. The crude reaction product was recrystallized in DCM/hexane. The product was collected as a bright yellow solid in 44% yield. $^1$H NMR (300 MHz, DMSO-d6): δ 8.05 (2 H, ArH, d, J = 8.3 Hz), 7.82 (2 H, ArH, d, J = 8.3 Hz), 7.65 (2 H, ArH, d, J = 8.9 Hz), 7.59 (1 H, oxazole H, s), 6.81 (2 H, ArH, d, J = 8.9 Hz), 2.97 (6 H, $CH_3$, s), 1.32 (12 H, pinacol ester $CH_3$, s); $^{13}$C NMR (75.5 MHz, DMSO-d6): δ 158.88, 152.48, 150.77, 135.43, 129.79, 125.72, 125.15, 121.53, 115.19, 112.51, 84.29, 40.19, 25.06; 11B NMR (96 MHz, DMSO-d6): δ 32.68; FTIR ν ($cm^{-1}$): 2976, 1610, 1356, 1325; HRMS (ES) m/z calculated for $C_{23}H_{28}N_2O_3B$: $[M+H]^+$ 391.2193, found 391.2185

## 4. NMR

**(E)-1-bromo-4-styrylbenzene** (300 MHz, $CDCl_3$)

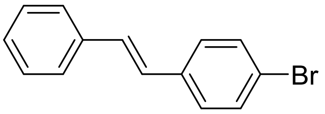


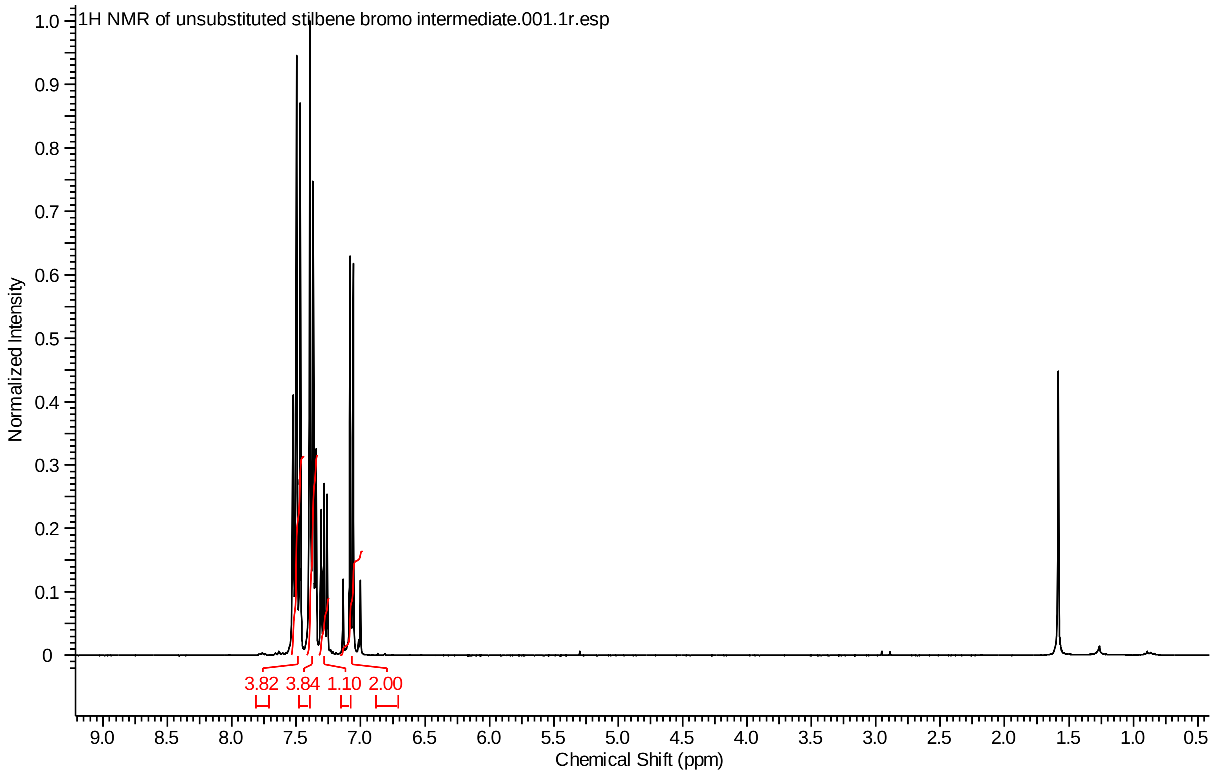

**(E)-1-bromo-4-styrylbenzene** (75.5 MHz, $CDCl_3$)

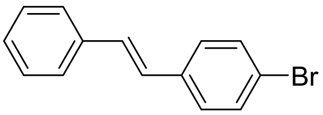


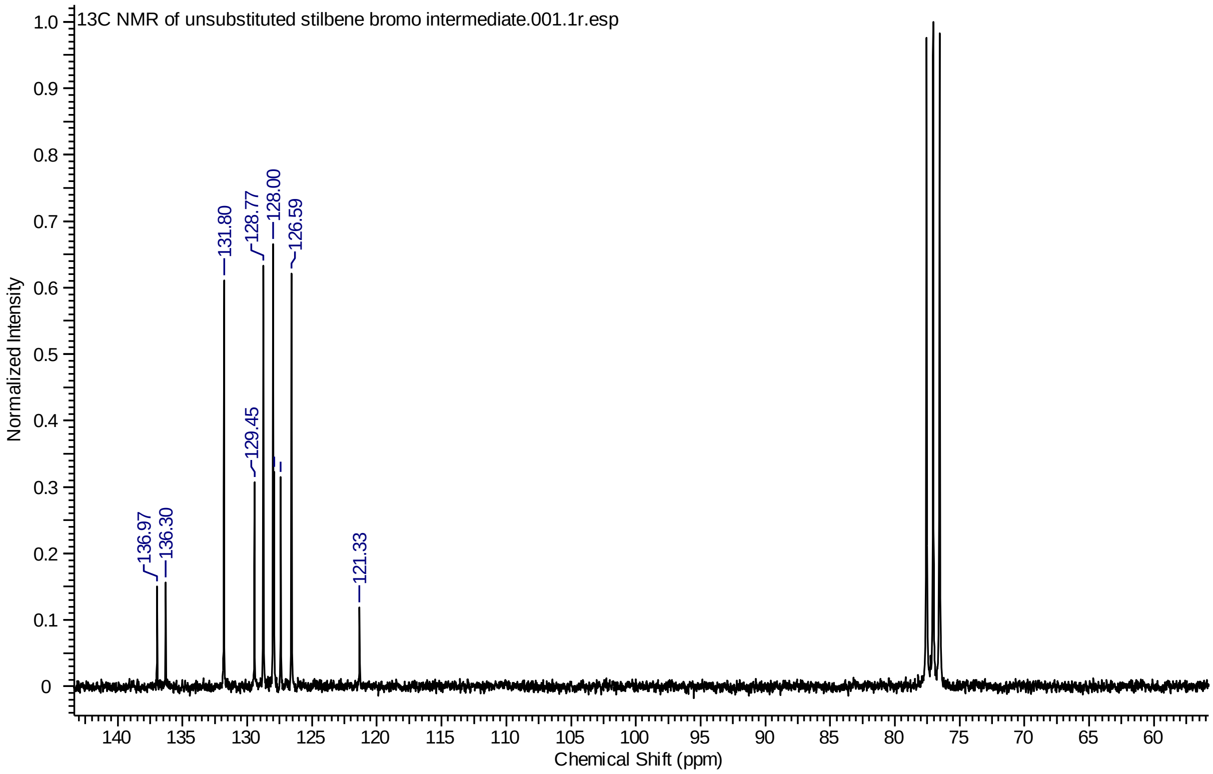

**(E)-4,4,5,5-tetramethyl-2-(4-styrylphenyl)-1,3,2-dioxaborolane** (300 MHz, $CDCl_3$)

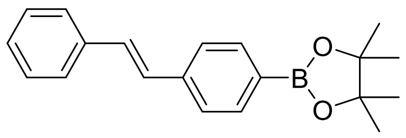


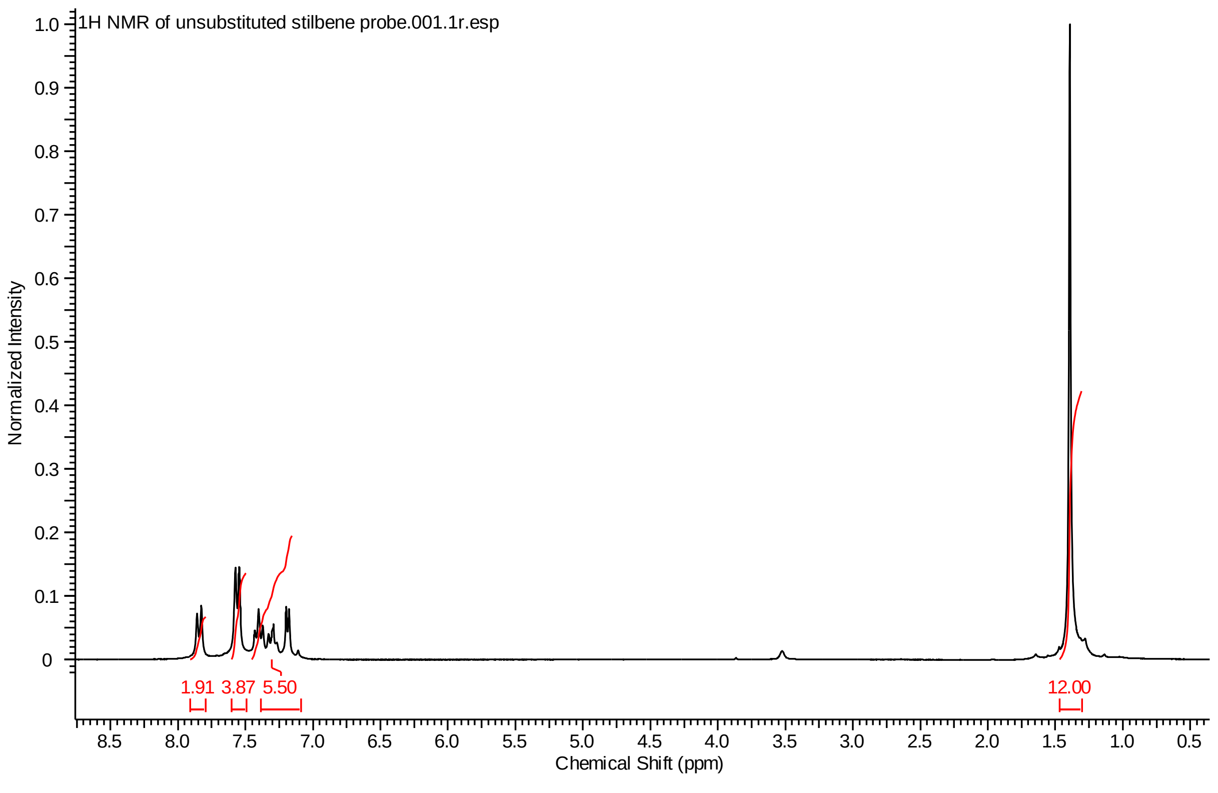

**(E)-4,4,5,5-tetramethyl-2-(4-styrylphenyl)-1,3,2-dioxaborolane** (75.5 MHz, $CDCl_3$)

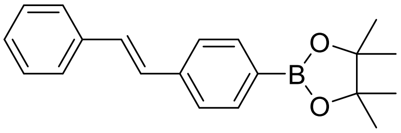


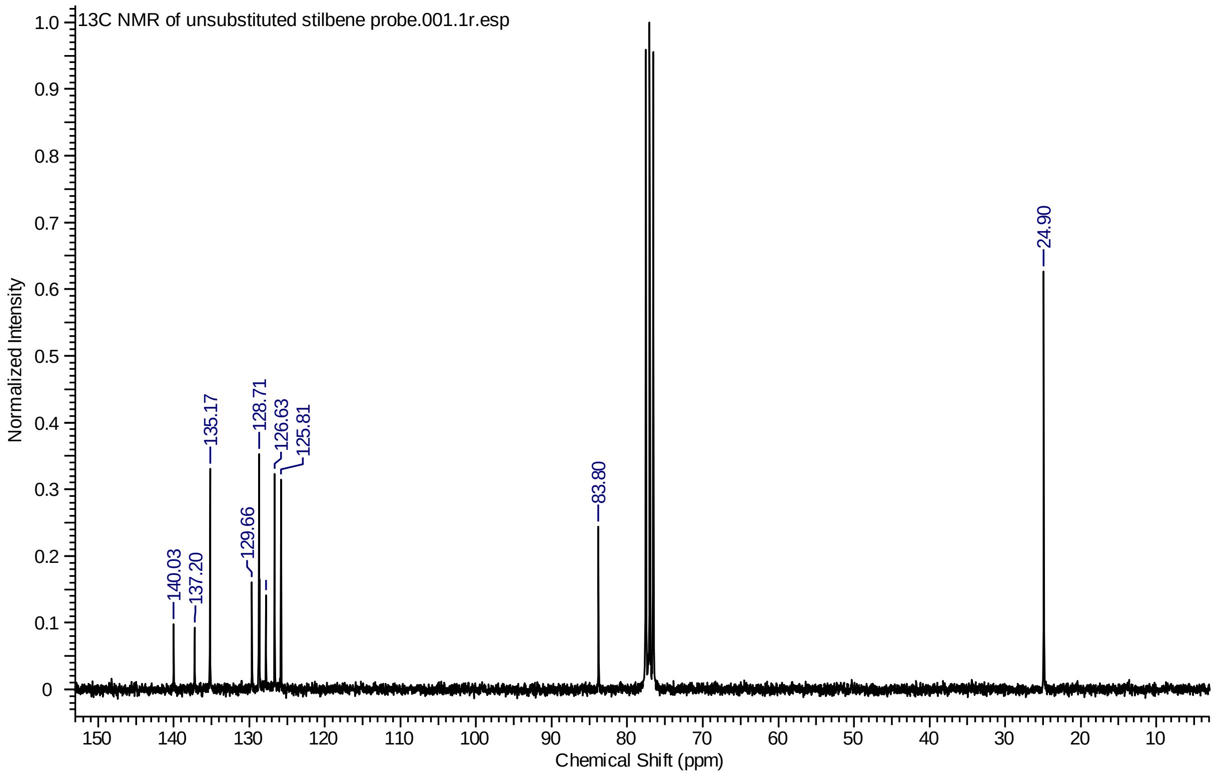

**(E)-4-(4-bromostyryl)-N,N-dimethylaniline** (300 MHz, $CDCl_3$)

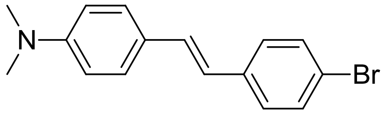


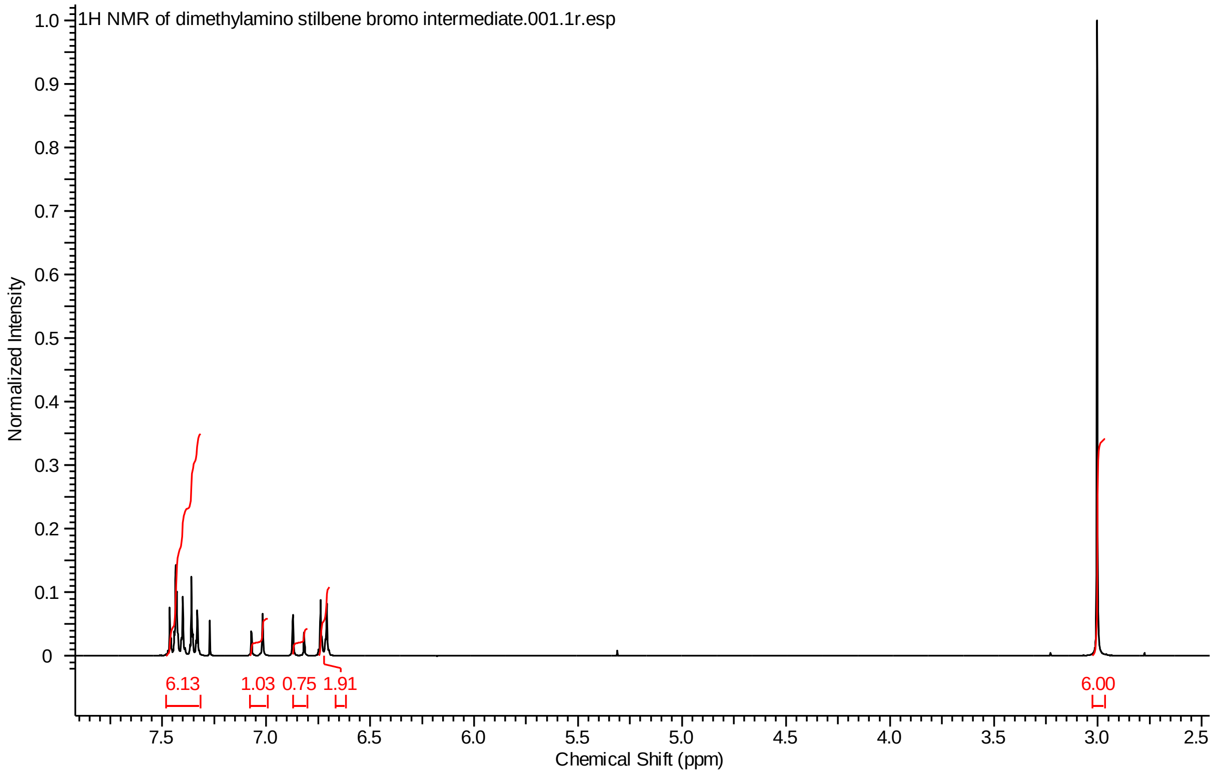

**(E)-4-(4-bromostyryl)-N,N-dimethylaniline** (75.5 MHz, $CDCl_3$)

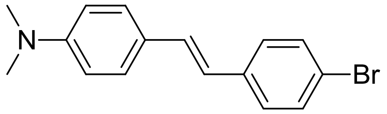


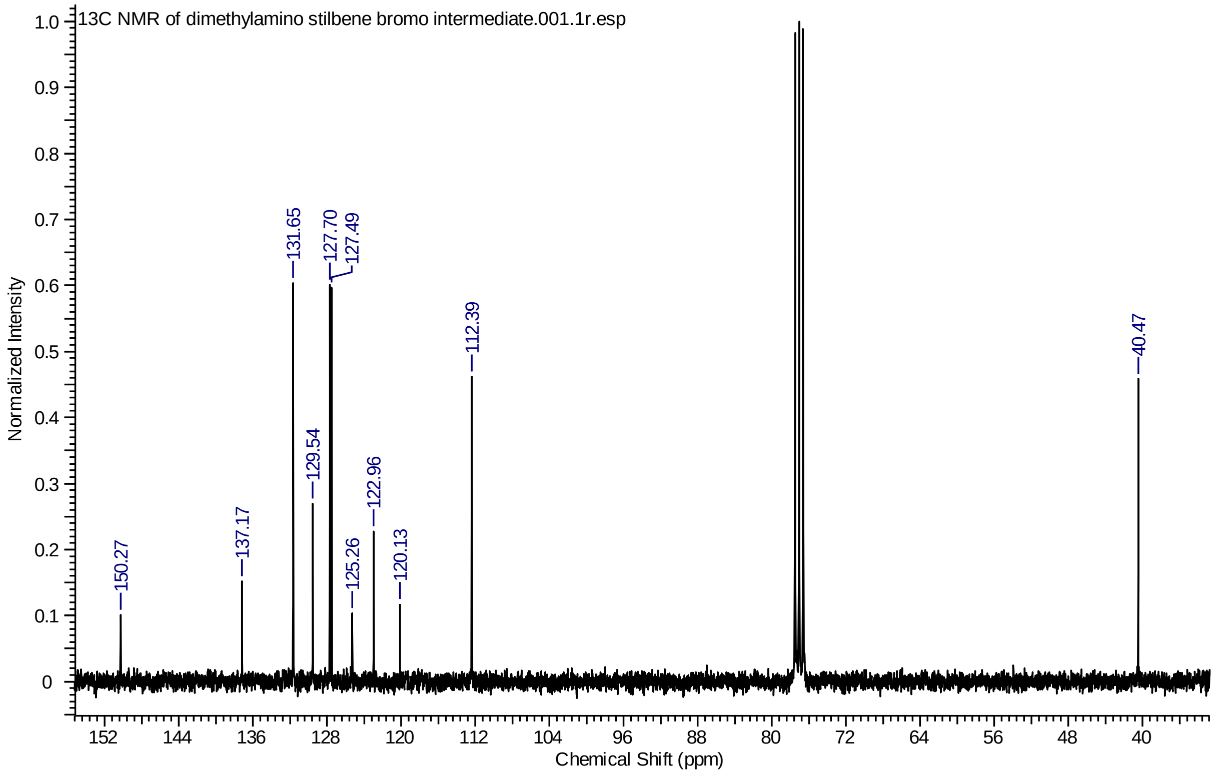

**(E)-N,N-dimethyl-4-(4-(4,4,5,5-tetramethyl-1,3,2-dioxaborolan-2-yl)styryl)aniline** (300 MHz, $CDCl_3$)

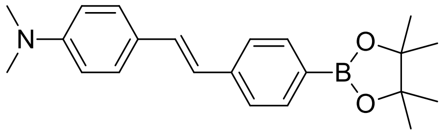

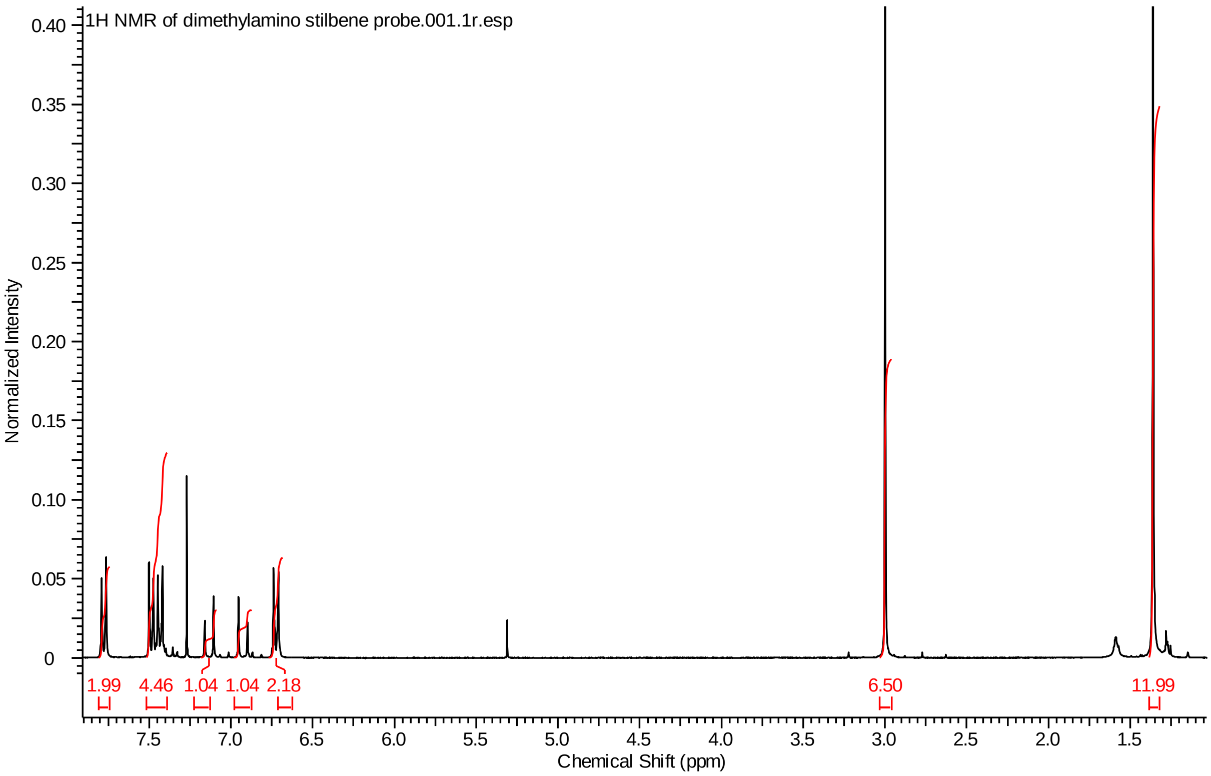

**(E)-N,N-dimethyl-4-(4-(4,4,5,5-tetramethyl-1,3,2-dioxaborolan-2-yl)styryl)aniline** (75.5 MHz, $CDCl_3$)

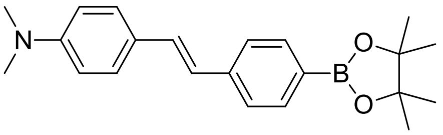


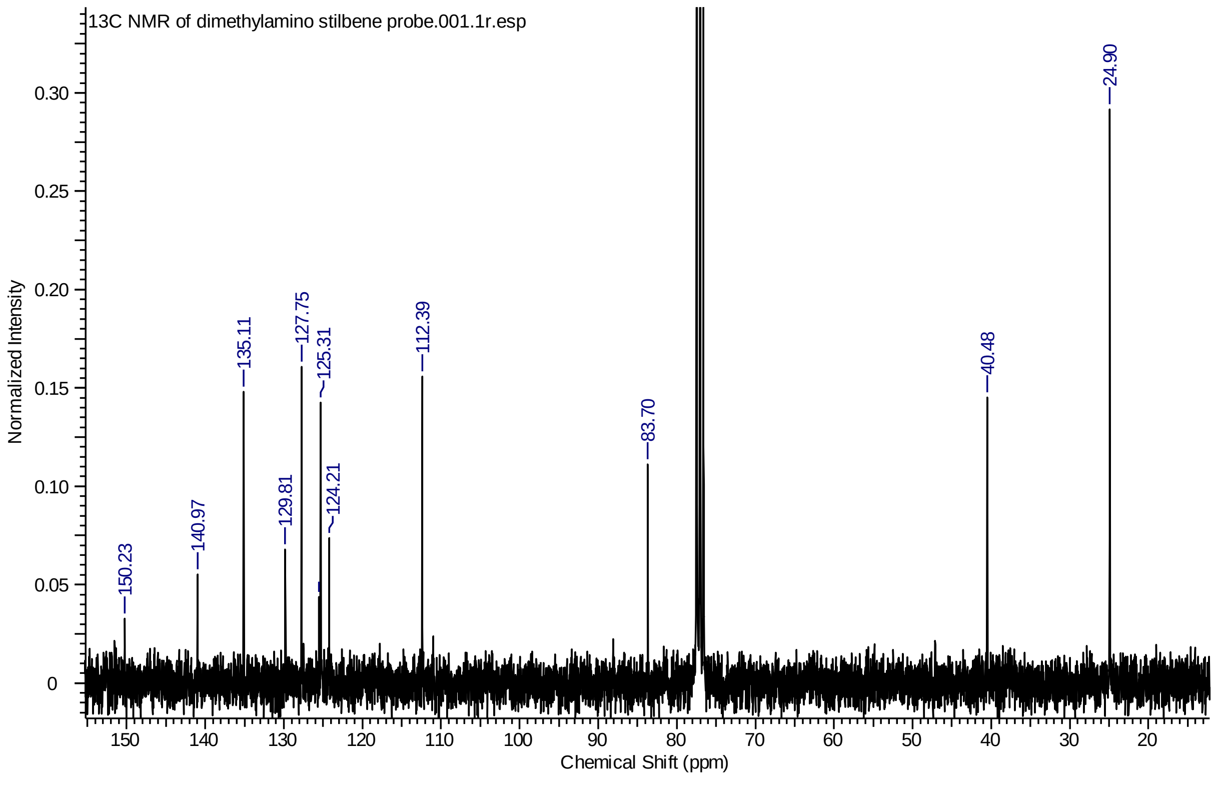

**(E)-1-bromo-4-(4-methoxystyryl)benzene** (300 MHz, $CDCl_3$)

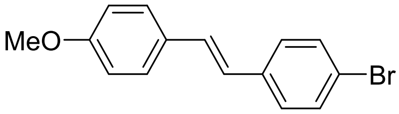


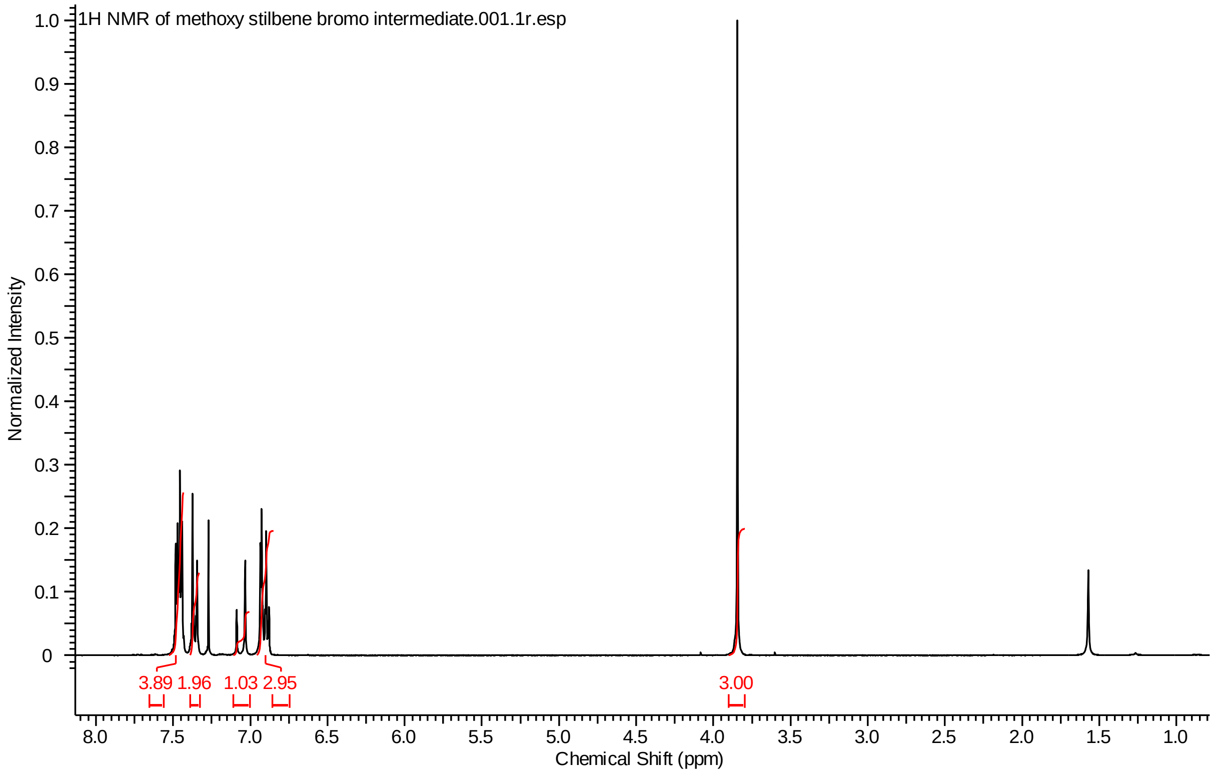

**(E)-2-(4-(4-methoxystyryl)phenyl)-4,4,5,5-tetramethyl-1,3,2-dioxaborolane** (300 MHz, $CDCl_3$)

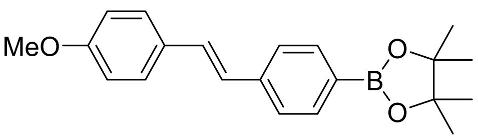


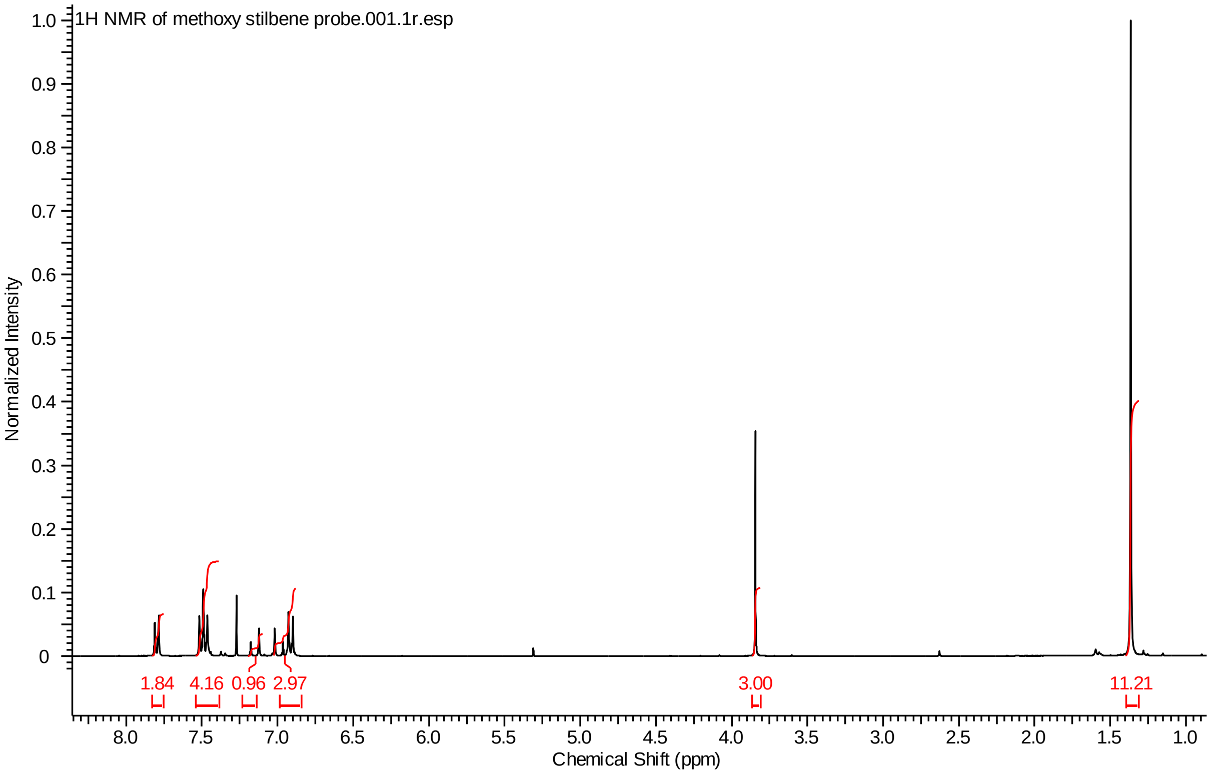

**(E)-2-(4-(4-methoxystyryl)phenyl)-4,4,5,5-tetramethyl-1,3,2-dioxaborolane** (75.5 MHz, $CDCl_3$)

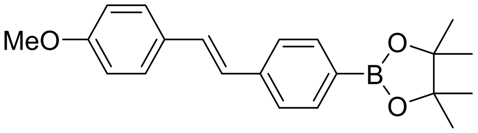


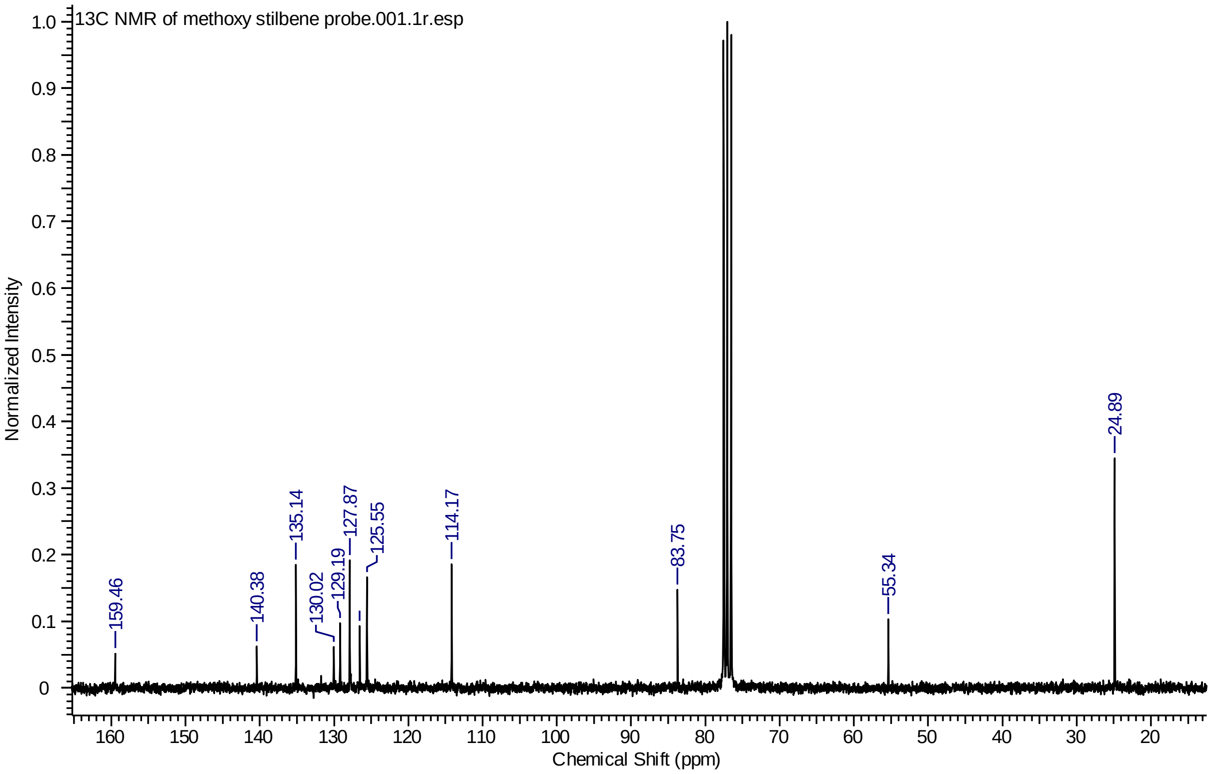

**(E)-4-(4-bromostyryl)benzonitrile** (300 MHz, $CDCl_3$)

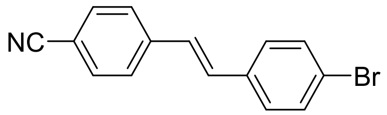


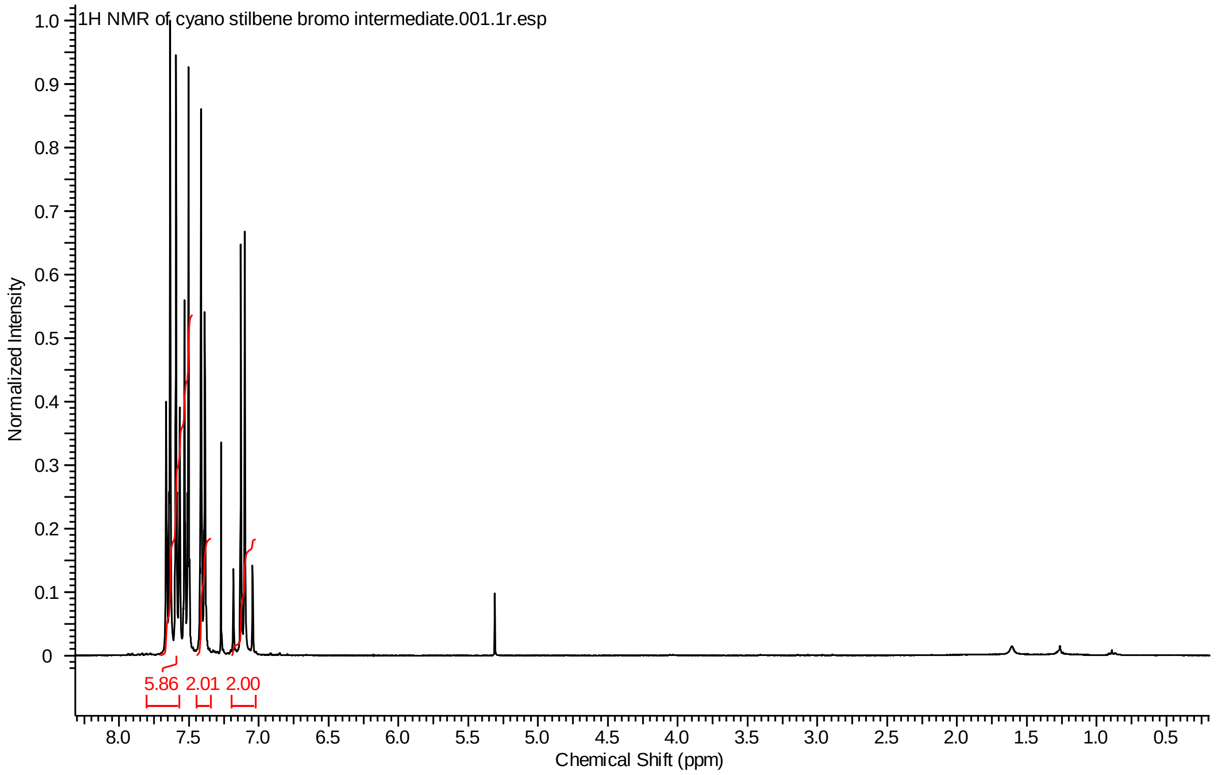

**(E)-4-(4-bromostyryl)benzonitrile** (75.5 MHz, $CDCl_3$)

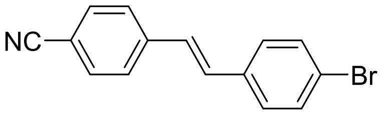


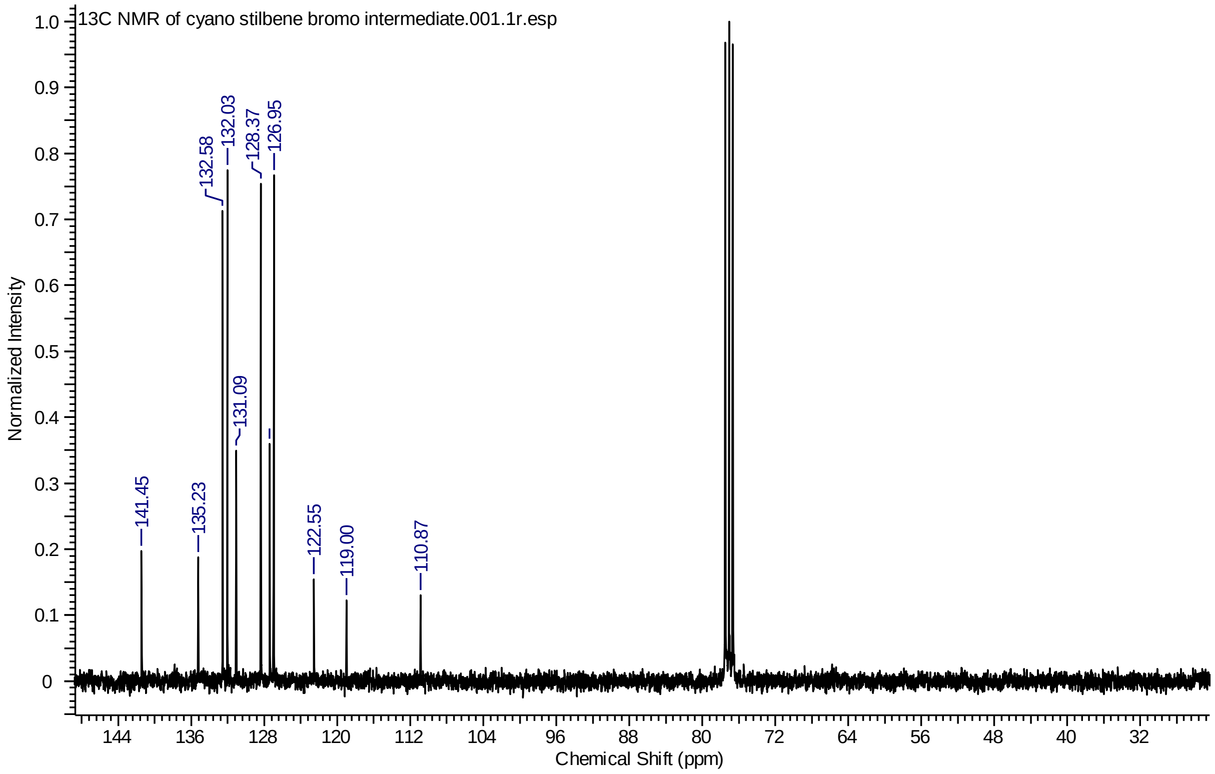

**(E)-4-(4-(4,4,5,5-tetramethyl-1,3,2-dioxaborolan-2-yl)styryl)benzonitrile** (300 MHz, $CDCl_3$)

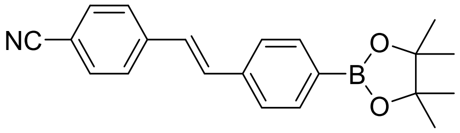


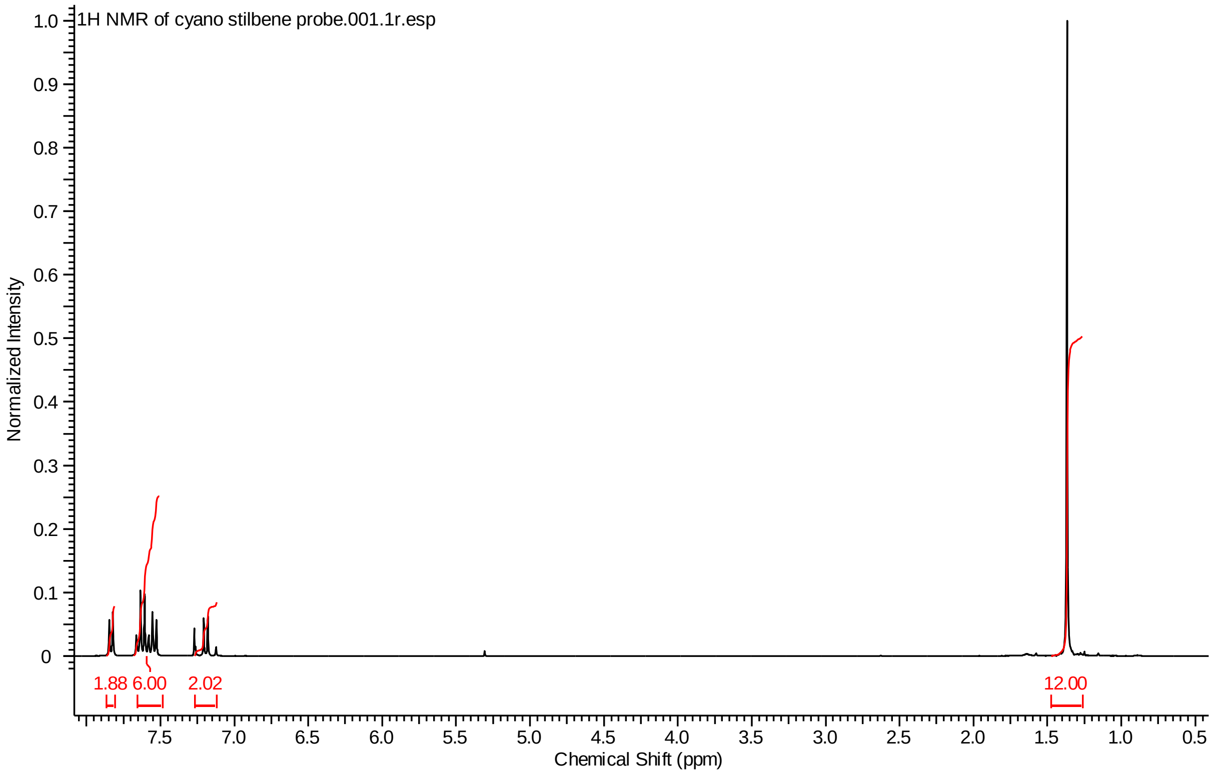

**(E)-4-(4-(4,4,5,5-tetramethyl-1,3,2-dioxaborolan-2-yl)styryl)benzonitrile** (75.5 MHz, $CDCl_3$)

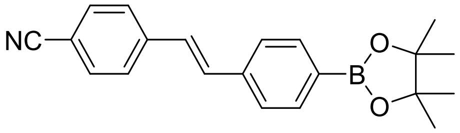

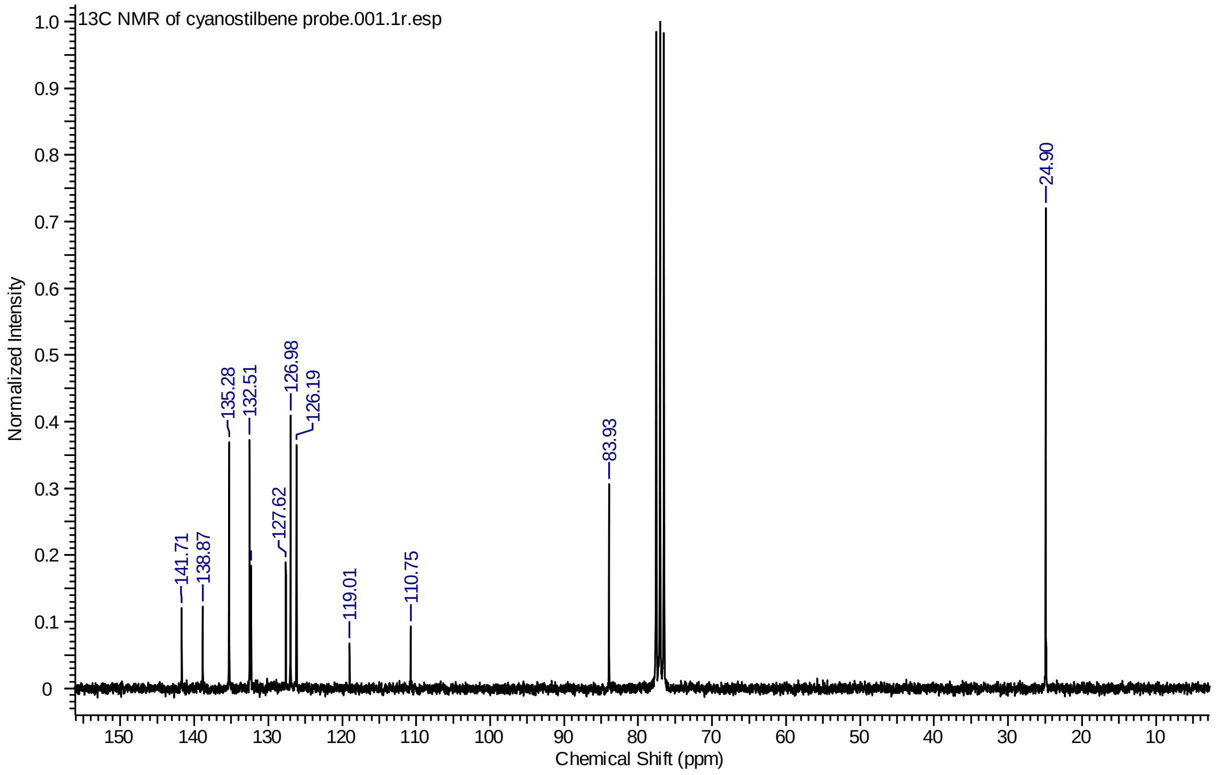


**(E)-4-(4-bromostyryl)-N,N-dimethylnaphthalen-1-amine** (250 MHz, $CDCl_3$)

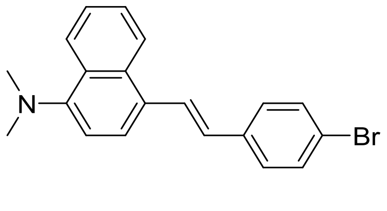

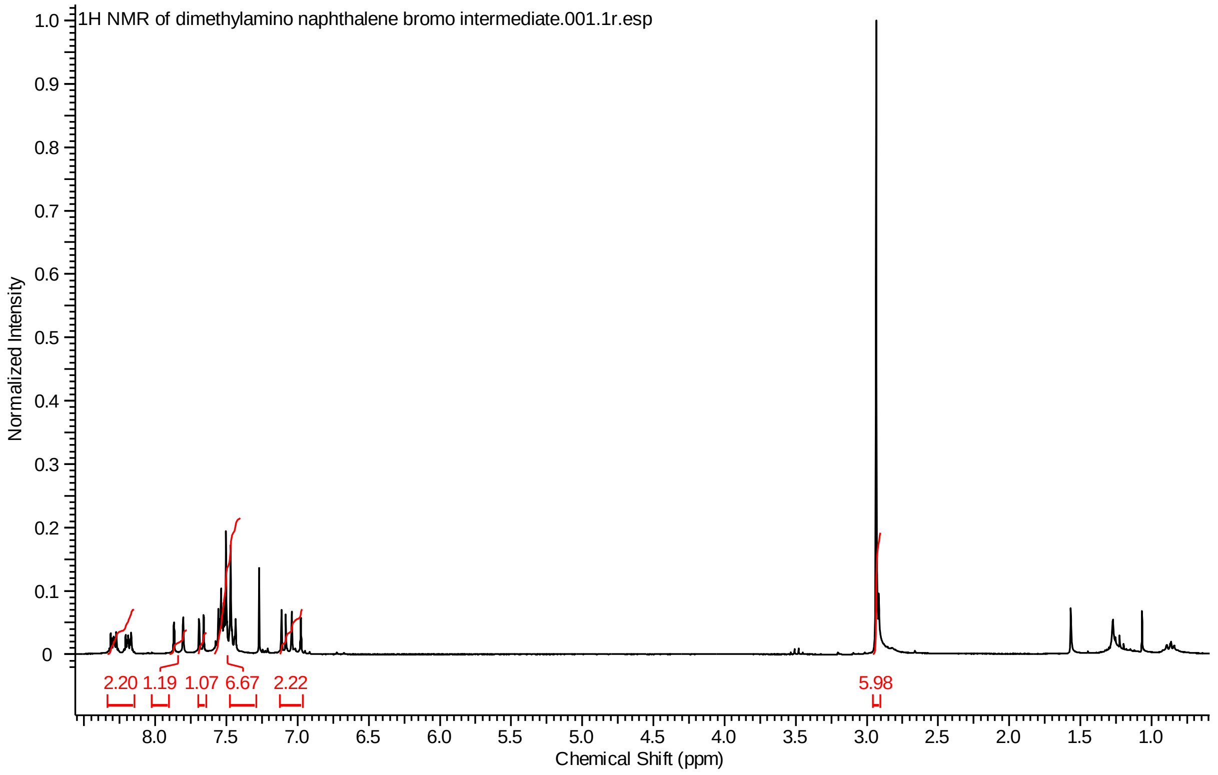


**(E)-4-(4-bromostyryl)-N,N-dimethylnaphthalen-1-amine** (75.5 MHz, $CDCl_3$)

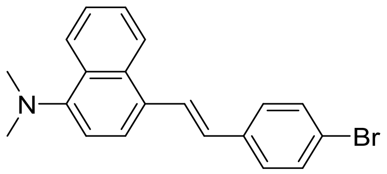

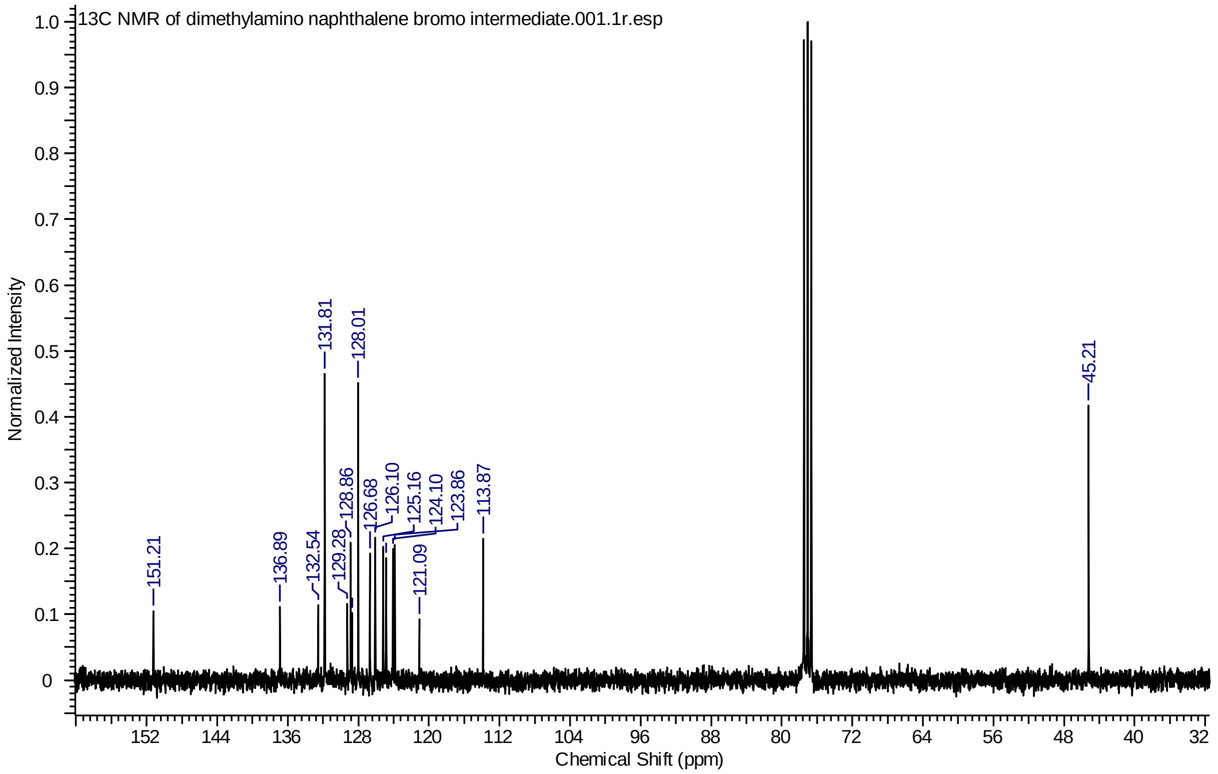


**(E)-N,N-dimethyl-4-(4-(4,4,5,5-tetramethyl-1,3,2-dioxaborolan-2-yl)styryl)naphthalen-1-amine** (300 MHz, $CDCl_3$)

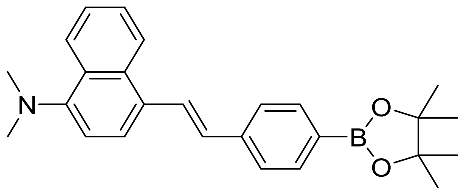

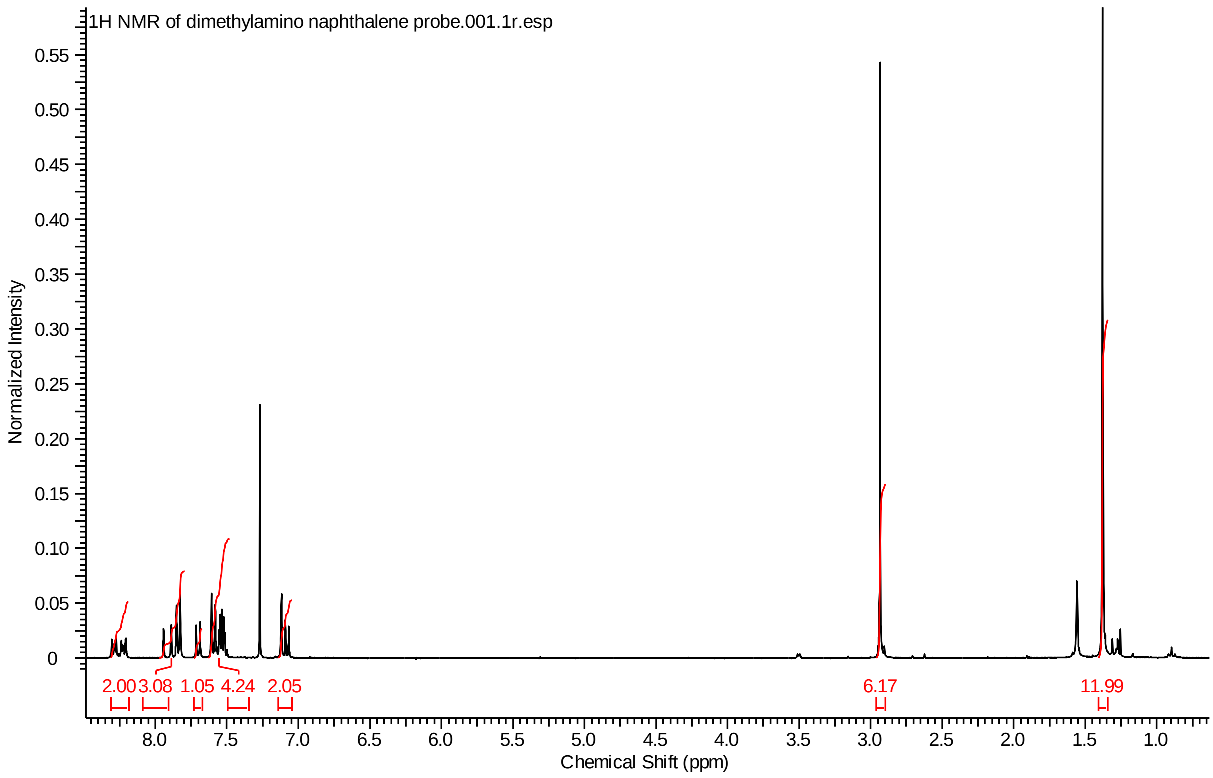


**(E)-N,N-dimethyl-4-(4-(4,4,5,5-tetramethyl-1,3,2-dioxaborolan-2-yl)styryl)naphthalen-1-amine** (75.5 MHz, $CDCl_3$)

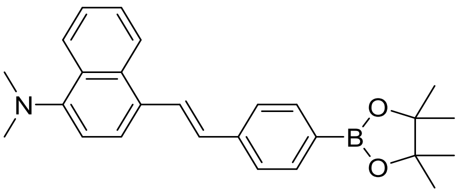

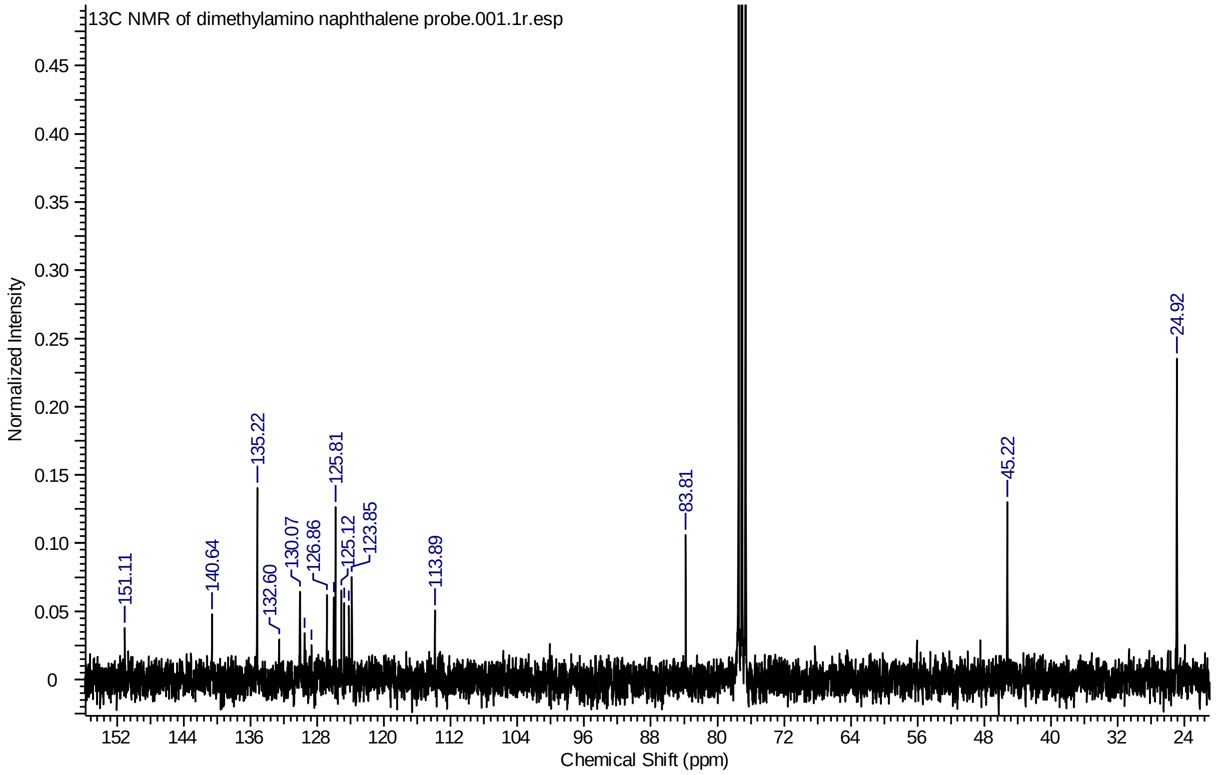


**4-Bromo-N-(2-(4-(dimethylamino)phenyl)-2-oxoethyl)benzamide** (300 MHz, $CDCl_3$)

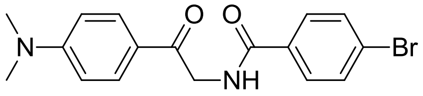


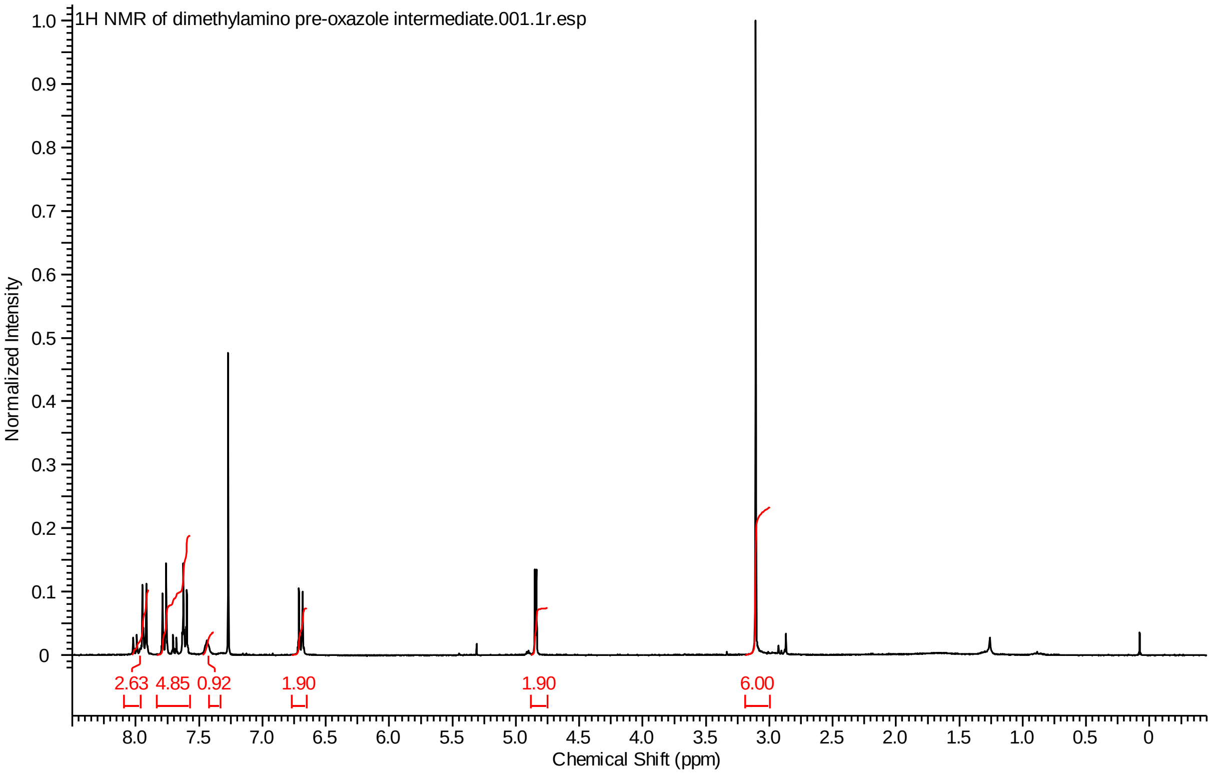


**4-(2-(4-bromophenyl)oxazol-5-yl)-N,N-dimethylaniline (**300 MHz, $CDCl_3$)

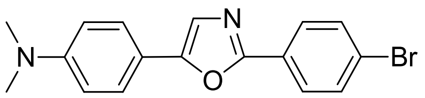


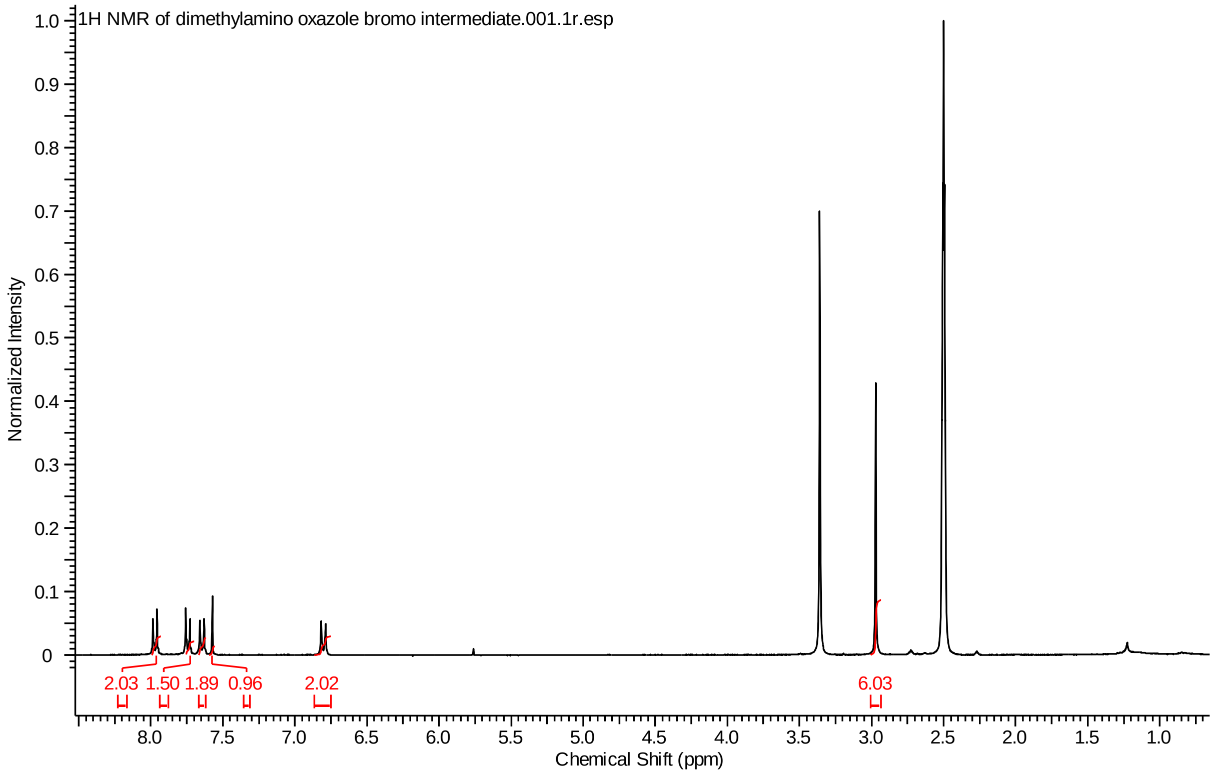


**N,N-dimethyl-4-(2-(4-(4,4,5,5-tetramethyl-1,3,2-dioxaborolan-2-yl)phenyl)oxazol-5-yl)aniline (**300 MHz, $CDCl_3$)

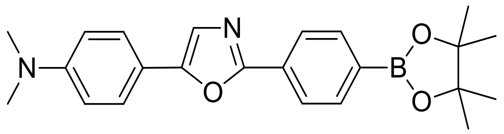


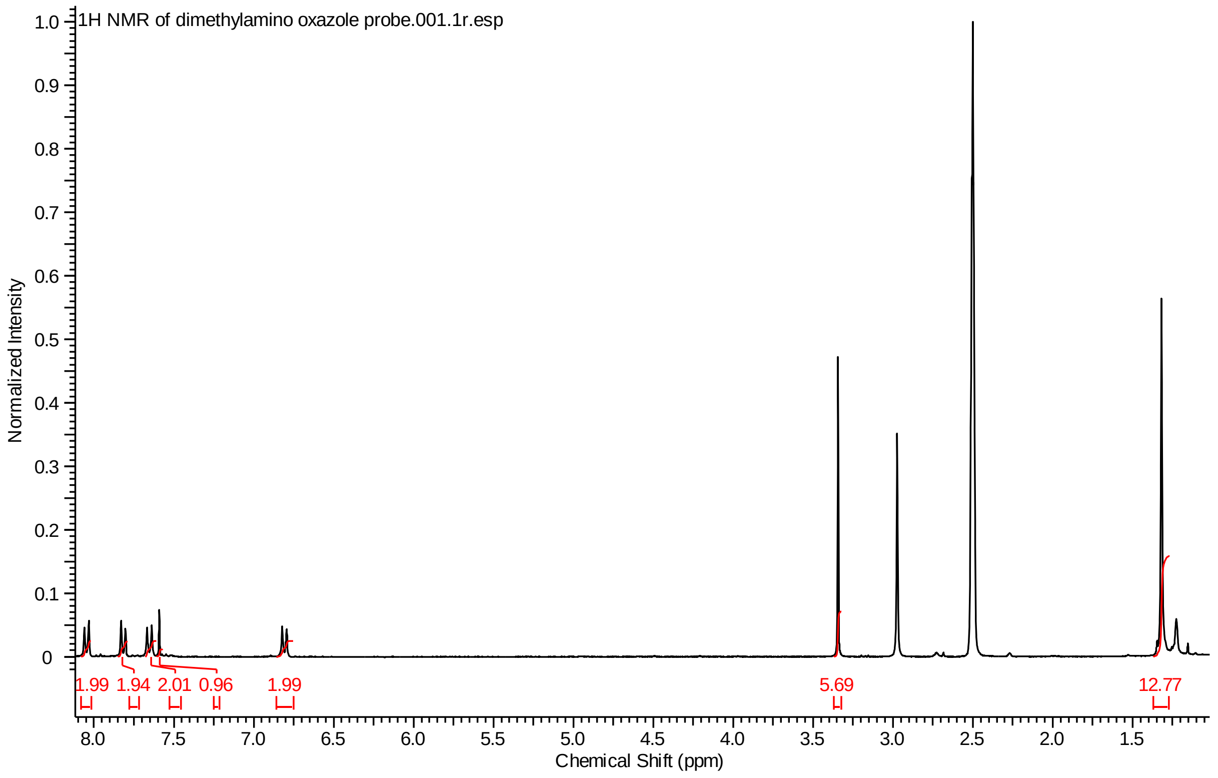


**N,N-dimethyl-4-(2-(4-(4,4,5,5-tetramethyl-1,3,2-dioxaborolan-2-yl)phenyl)oxazol-5-yl)aniline (**75.5 MHz, $CDCl_3$)

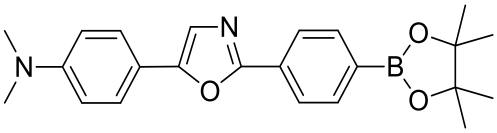


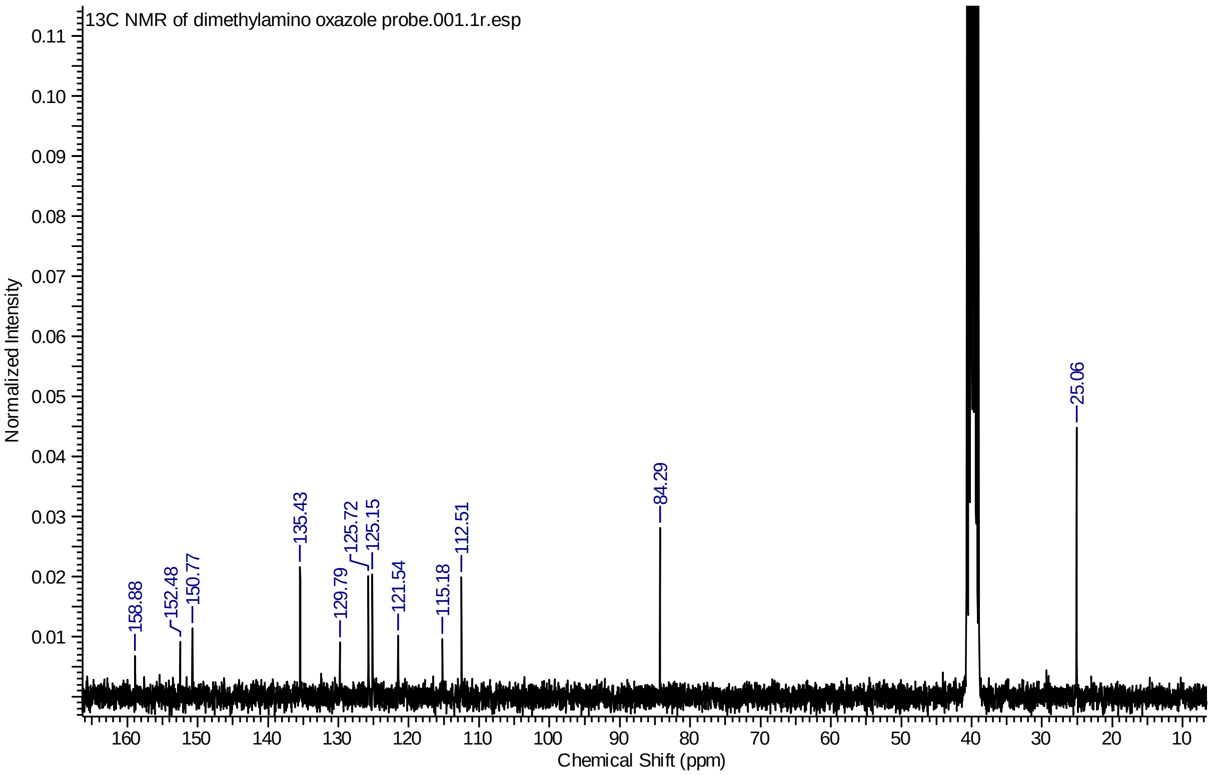


## 5. References

(1) Gigante, B.; Esteves, M. A.; Pires, N.; Davies, M. L.; Douglas, P.; Fonseca, S. M.; Burrows, H. D.; Castro, R. A. E.; Pina, J.; de Melo, J. S. Synthesis, spectroscopy, photophysics and thermal behaviour of stilbene-based triarylamines with dehydroabietic acid methyl ester moieties. *New Journal of Chemistry* **2009,** *33* (4), 877.

(2) Das, B. C.; Mahalingam, S. M.; Das, S.; Hosmane, N. S.; Evans, T. Synthesis of Pinacolylboronate-Substituted Stilbenes and their application to the synthesis of boron capped polyenes. *Journal of Organometallic Chemistry* **2015,** *798*, 51.
